# A versatile route to edge-specific modifications to pristine graphene by electrophilic aromatic substitution

**DOI:** 10.1007/s10853-020-04662-y

**Published:** 2020-05-09

**Authors:** Philippa M. Shellard, Thunyaporn Srisubin, Mirja Hartmann, Joseph Butcher, Fan Fei, Henry Cox, Thomas P. McNamara, Trevor McArdle, Ashley M. Shepherd, Robert M. J. Jacobs, Thomas A. Waigh, Sabine L. Flitsch, Christopher F. Blanford

**Affiliations:** 1 Department of Chemistry, University of Manchester, Oxford Road, Manchester, M13 9PL UK; 2 Manchester Institute of Biotechnology, University of Manchester, 131 Princess Street, Manchester, M1 7DN UK; 3 Department of Materials, University of Manchester, Oxford Road, Manchester, M13 9PL UK; 4 Biological Physics, Department of Physics and Astronomy, University of Manchester, Oxford Road, Manchester, M13 9PL UK; 5 Photon Science Institute, University of Manchester, Alan Turing Building, Oxford Road, Manchester, M13 9PL UK; 6 Chemical Research Laboratory, Department of Chemistry, University of Oxford, 12 Mansfield Road, Oxford, OX1 3TA UK

## Abstract

**Electronic supplementary material:**

The online version of this article (10.1007/s10853-020-04662-y) contains supplementary material, which is available to authorized users.

**Figure:**
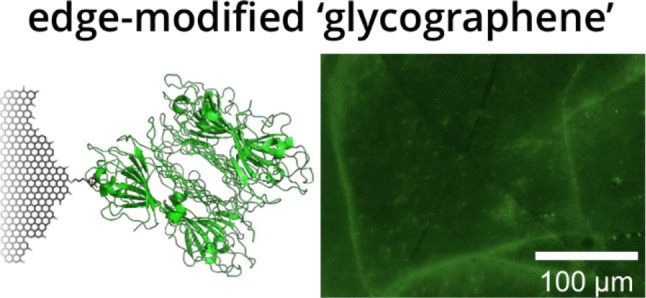
Electrophilic aromatic substitution produces edge-specific modifications to CVD graphene and graphene nanoplatelets that are suitable for specific attachment of biomolecules

## Introduction

Graphene, a “two-dimensional” material made of *sp*^2^-hybridised carbon, is an attractive platform for nanomedicine, including drug delivery [1], theranostics [2], non-viral gene transfer [3], regenerative medicine [4, 5], sensors [6], and bioelectronics [7], because of its unique combination of properties including high carrier mobility [8], high yield strength [9, 10], and facile chemical modification [11]. Chemical modifications are useful for tuning graphene’s solvent dispersibility and for providing chemically reactive attachment points for further modifications, such as bonding to a matrix in a nanocomposite [12] or the attachment of biomolecules [13, 14].

Most existing covalent functionalisation of graphene family nanomaterials is based on grafting molecules through oxygen-containing functional groups of graphene oxide (GO), followed by chemical or thermal reduction to obtain reduced graphene oxide (rGO) [15, 16]. Graphene oxide (GO) is an oxidised, exfoliated form of graphite with a prevalence of oxygen-containing functional groups (carboxyl, hydroxyl and epoxide) on its exfoliated sheets. GO has become widely applied because it provides hydrophilic functional groups that allow it to form a stable dispersion in aqueous and polar solvents [17]. The oxidation process, however, generates defects on the GO sheets which disrupt *π*–*π* conjugation, leading to the loss of mechanical strength, as well as reduced electrical and thermal conductivity [18, 19]. Young’s modulus for GO is five times lower than that of single-layer graphene [20], and even the most conductive rGO has a carrier mobility ~ 10^3^ times lower than pristine graphene [21]. GO-based materials are not well suited to applications that require on robust mechanical or electronic properties.

Functionalisation of pristine graphene can be achieved by both covalent and non-covalent interactions [15]. Pristine graphene has been predominantly modified through non-covalent methods because it possesses an extended *π* system which forms attractive hydrophobic and *π*–*π* interactions with aromatic molecules [15, 22]. Pristine graphene has been coated with pyrene [23, 24], pyridinium tribromide [25], triphenylene [26], and coronene [27], as well as biological surfactant molecules such as phospholipids and cholesterol [28]. Although these modifications overcome graphene’s problem of poor dispersibility in buffered aqueous media, surfactant molecules remain adsorbed on the surface and influence the biocompatibility [28] and conductivity [29] of the product.

Covalent modification of pristine graphene can be achieved through free radical addition to *sp*^2^ carbons on the basal plane using diazonium salts [30–37] or benzoyl peroxide [38] to form radicals. Graphene has also been covalently linked to dienes and dienophiles using the Diels–Alder reaction [39–47]. This reaction generally disrupts graphene’s extended *π* system and so degrades the product’s conductivity due to the conversion of *sp*^2^ carbons to *sp*^3^ carbons [15, 22, 33, 48], but some exceptions have been discussed based on DFT calculations [48, 49].

Edge-selective covalent functionalisation minimises chemical reactions on graphene’s basal plane and the attendant deterioration of engineering properties. Ball milling graphite in the presence of gases or gas mixtures has been reported to produce edge-specific functionalisation [50]. The technique produces reactive species (e.g. radicals, cations, and anions) that react with defects introduced in the graphite as it is being broken down by ball milling. The process has been used to introduce sulphonic acids [50], carboxylic acids [50–52], phosphonic acids [53], and halogens to the graphite [54]. However, ball milling process can generate a violent sparking reaction caused by active carbon species, metallic debris, and moisture in the air [55]. Ball milling also can introduce metallic residues from the balls, which require acidic work-up to remove [55]. An alternative method for achieving the edge-specific functionalisation of pristine graphene and GO is Friedel–Crafts acylation [12, 56, 57], where an acyl chloride with a Lewis acid such as AlCl_3_ is traditionally used. Benzoic acid derivatives with polyphosphoric acid and phosphorus pentoxide have been used for milder graphene acylations [12, 56, 57]. However, the presence of the carbonyl group which arises from Friedel–Crafts acylation may slow electron transfer between the graphene sheet and groups attached to its edges [58–60]. Other edge-functionalised graphene-type materials have been made from bottom-up methods such as Suzuki coupling of bromine-containing polyphenylene precursors followed by intramolecular oxidative cyclodehydrogenation [61]. Furthermore, edge-selective functionalisation of graphene monolayers treated by oxygen plasma can also be achieved electrochemically [52].

The current work extends the top-down method of electrophilic aromatic substitution to create five distinct types of edge-modified graphene with no intermediate carbonyl moiety. Our modifications significantly modified the graphene nanoparticles’ dispersibility. The synthetic utility of the directly attached reactive moieties was demonstrated by creating a “glycographene” through radical addition of allyl mannoside to edge-thiolated graphene. Chemical modifications were confirmed by FT-IR and XPS. Edge localisation was visualised on modified CVD graphene samples by scanning electron microscopy of gold nanoparticles attached to thiol groups, epifluorescence microscopy of a fluorescently tagged lectin–glycographene bioconjugate, and quenched stochastic optical reconstruction microscopy (qSTORM) [62–66], a super-resolution technique, of thiol-reactive fluorophores on edge-thiolated graphene.

## Experimental methods

### Materials

Graphene/graphite nanoplatelets (GNPs; grades C750, C300, and M25) were purchased from XG Sciences (Lansing, MI, USA). The C750 and C300 grades have nominal specific surface areas of 750 m^2^ g^−1^ and 300 m^2^ g^−1^, respectively, corresponding to 4–5 graphene layers for C750 and 8–9 layers for C300. The batches used in this work had BET specific surface areas of 794.9 ± 1.6 m^2^ g^−1^ and 268 m^2^ g^−1^. The M25 grade has a nominal lateral dimension of 25 µm. The specific surface area specified by the manufacturer is 120–150 m^2^ g^−1^, corresponding to about 17–22 graphene layers; the batch used in this work had a BET specific surface area of 55 m^2^ g^−1^. On receipt, the GNPs were portioned into 50-ml Falcon tubes and sealed with Parafilm.

Monolayer graphene produced by chemical vapour deposition (CVD) on copper was provided by 2-DTech (Manchester, UK) and transferred as ~ 1 cm^2^ sheets to a silicon wafer covered with 300 nm thermal oxide. Before use, CVD graphene was stored in a covered Petri dish under ambient conditions.

Graphene dispersions were also prepared by ultrasonic exfoliation of natural graphite flakes (Branwell Graphite, Ltd, Grade 2369). Following the method of Hernandez [67], 2 g graphite was sonicated in 500 ml *N*-methyl-2-pyrrolidone (NMP) at 37 Hz for 48 h in an ultrasonic bath (Elmasonic PH750EL). Remaining graphite was removed by cetrifugation (3 × 20 min at 4000 rpm). The supernatant was a graphene dispersion (ca. 0.4 g l^−1^). Graphene reaction dispersions were vacuum filtered through 0.02-µm Whatman Anodisc membrane filters to create graphene laminates. For analysis, samples were dried as laminates on the filter membrane or re-dispersed in water by sonication and freeze-dried (HETO PowderDry LL1500 Freeze Dryer, Thermo Electron Corporation). For long-term storage, the samples were kept in a desiccator at room temperature.

*N,N*-dimethylformamide (DMF, 99%) and methanol were purchased from Fisher Scientific, UK. Sodium borohydride (≥ 96%), iodine (≥ 99.8%) and triphenylphosphine (99%) were purchased from Sigma-Aldrich. Raney nickel catalyst (50% in water) and chlorosulphonic acid (1.75 g cm^−3^, ≥ 98%) were purchased from Merck Chemicals. All chemicals were used as supplied. Water was purified to a resistivity of 18.2 MΩ cm at 25 °C (Milli-Q).

SnakeSkin dialysis tubing (10 kDa MWCO, 35 mm dry ID) was purchased from Thermo Scientific. Disposable folded capillary cells for Zetasizer Nano Series were purchased from Malvern.

### Synthesis of edge-modified graphene

#### Modifications to graphene nanoplatelets

**Scheme 1:**
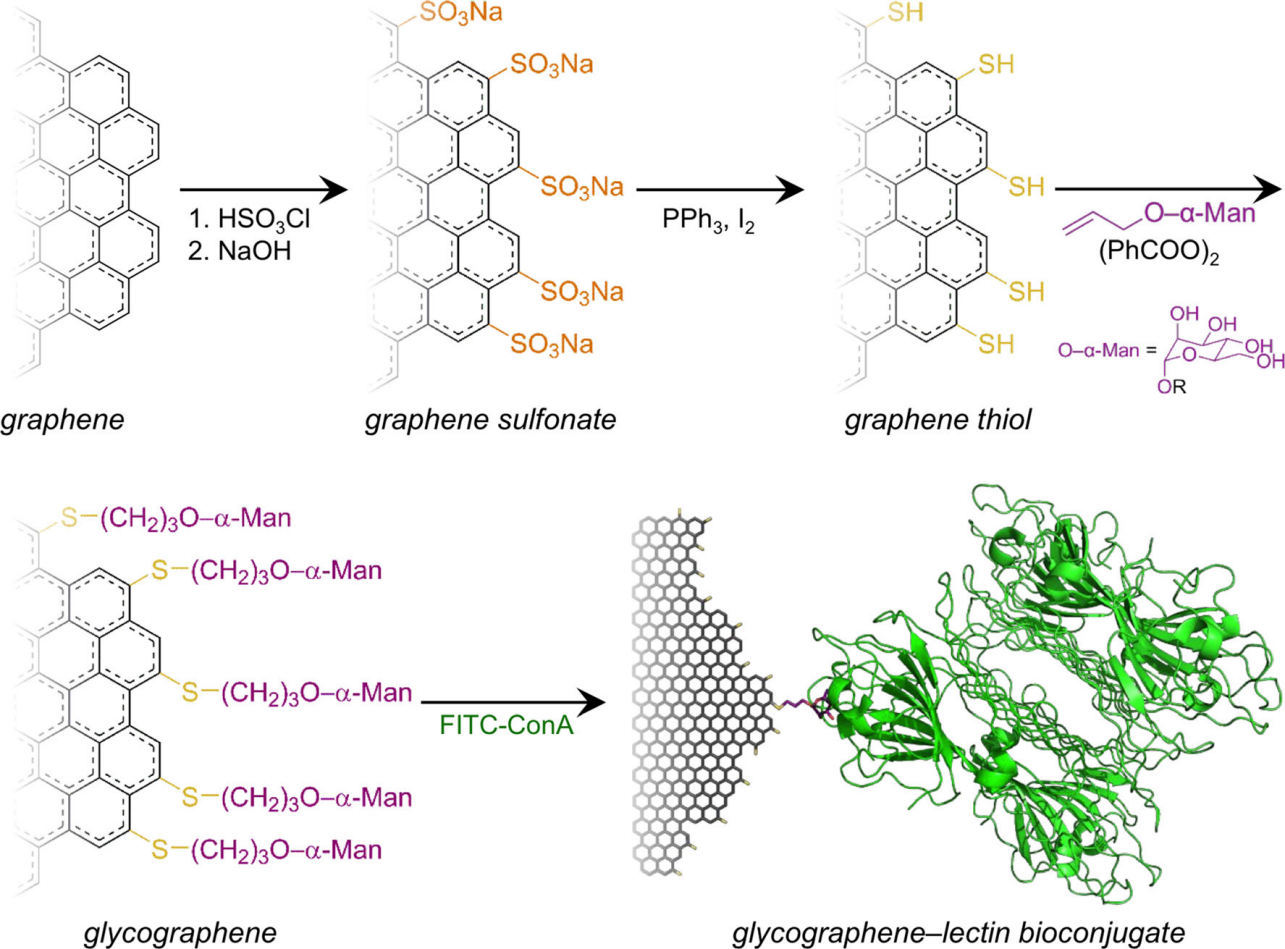
Synthesis of graphene sulphonate (G–SO_3_^−^) by electrophilic aromatic substitution, followed by its subsequent reduction to a thiol-containing form (G–SH), coupling to allyl mannoside, and selective binding of a ConA lectin tetramer (PDB: 5CNA)

Sulphonated graphene (G–SO_3_^−^, Scheme 1) was synthesised by suspending 500 mg GNPs in 10 ml neat chlorosulphonic acid at room temperature. The temperature was increased to 100 °C, and the suspension was stirred for 20 h. After cooling, the reaction mixture was poured into ice water and then neutralised using aqueous NaOH and universal pH paper. The material was dialysed by pouring ~ 100 ml of neutralised suspension into dialysis tubing and suspending it in ~ 2 l stirred deionised water. The water was changed 3 times at intervals of at least 8 h. The deionised suspension was filtered, resuspended in water, and then freeze-dried.

Thiolated graphene (G–SH, Scheme 1) was synthesised by suspending 50 mg of graphene sulphonate (G-SO_3_) in 30 ml of toluene and sonication under N_2_ atmosphere for 15 min. Then, the reaction flask was connected to a condenser with nitrogen flow. 2.5 g of triphenylphosphine and 200 mg iodine were added into the mixture that was left stirring at 80 °C for 21 h. The product was filtered using vacuum filtration and 0.45-μm HV membrane filter and washed with toluene, acetone, 0.1 M sodium thiosulphate solution, and deionised water, followed by freeze-drying.

#### Modifications to CVD graphene

Before modification, samples were soaked in acetone for 30 min to remove residual PMMA and dried and then transferred to 20% v/v chlorosulphonic acid solution in DMF for 30 s at room temperature. Samples were then washed with deionised water and acetone to remove any remaining acid and hydrolysed by immersing in pH 10 NaOH solution for 15 min. These were washed with water and acetone and dried in a nitrogen stream, leaving CVD graphene sulphonate.

Thiolated CVD graphene was produced by immersing a sulphonated sample for 15 min in 10 ml anhydrous toluene containing 10 mM triphenylphosphine and 0.6 mM iodine. A nitrogen atmosphere was maintained throughout the procedure by flushing with a strong flow of dry house nitrogen. The sample was removed and washed with distilled water and acetone and dried as with sulphonate. Thiolated graphene that was not immediately subsequently functionalised was kept under vacuum to prevent thiol oxidation.

Gold nanoparticles (Aldrich, 20 nm diameter in 0.1 M phosphate-buffered saline, ~ 6 × 10^11^ particles ml^−1^) were bound to thiolated CVD graphene. The nanoparticle solution was diluted tenfold in phosphate-buffered saline (PBS pH 7.4, 0.01 M phosphate, 0.138 M NaCl, 2.7 mM KCl). A sample of modified CVD graphene was immersed in 10 ml of the diluted nanoparticle suspension overnight at ambient temperature. The sample was rinsed with water and acetone and dried in a nitrogen gas stream and stored at room temperature.

Glycographene (Scheme 1) was made by immersing thiolated CVD graphene in ethanol containing 150 mg (682 µmol) allyl mannoside and one spatula benzoyl peroxide overnight at 65 °C. The CVD graphene sample was rinsed with ethanol, water and acetone, dried in a nitrogen gas stream, and stored at ambient temperature. Concanavalin A labelled with fluorescein isothiocyanate (FITC-ConA) was dissolved in binding buffer (20 mM Tris, 500 mM NaCl, 1 mM CaCl_2_, 1 mM MgCl_2_, pH 7.2) in a glass vial wrapped in aluminium foil. The CVD glycographene sample was added to the vial and incubated at 37 °C for 2 h. The sample was rinsed with water and dried in a nitrogen gas stream.

### Characterisation

Raman spectra were recorded from 100 to 3200 cm^−1^ using a Renishaw inVia, with a 633-nm excitation laser set to 10% (0.89 mW) power or a 514-nm laser (0.176 mW). The energy resolution was 0.3 cm^−1^. Raman shifts were calibrated by setting the G (E_2g_) peak from silicon to 1581 cm^−1^ [68, 69]. The use of two excitation wavelengths led to positive shifts in the D and 2D peak positions when using the higher energy green excitation [69, 70]. Raman maps of modified CVD graphene samples were recorded on a Renishaw InVia with a 633-nm laser through a 50 × Leica NPLAN EPI objective (NA = 0.75). Spectra were acquired from 1000 to 3000 cm^−1^ every 0.5 µm (25 accumulations, 20 s exposure, 1%/0.016 mW laser power). Data were processed using WiRE 4.2 software to zero the baseline and remove cosmic rays. Nonlinear fits (SI) were applied to the peaks following the methods of Puech et al. [71]. Domain sizes were estimated from the relative intensity of the *D* band (i.e. *I*_*D*_/*I*_*G*_) using the Tuinstra–Koenig relationship ($L_{a}^{\text{TK}}$, Eq. S1) [72], the relative area of the *D* band (i.e. *A*_*D*_/*A*_*G*_) using the relationship from Cançado et al. ($L_{a}^{\text{A}}$, Eq. S2) [73], and the HWHM of the D peak ($L_{a}^{\text{HWHM}}$, Eq. S3) [74]. The average spacing between point defects (*L*_*D*_) was estimated using the expression from Lucchese et al. (Eq. S4) [75].

X-ray photoelectron spectra (XPS) were acquired using a Kratos Axis Ultra with an Al Kα source (1486.6 eV) operated at 15 kV and 10 mA. The pressure of the vacuum chamber was below 5 × 10^−8^ mbar during measurements. Peaks were fit using CasaXPS with Shirley background correction. The C 1*s* energy was calibrated by fixing the binding energy for the *sp*^2^ component to 284.5 eV. The C 1*s* peak was fit as five components summarised in Table 1, which consistently fit the data adequately for all samples [76]. Uncertainty estimates in the *sp*^3^:*sp*^2^ C ratio were from Monte Carlo simulations within CasaXPS. Fits to 2*p*_1/2_ and 2*p*_3/2_ peaks were constrained to have identical FWHM values and an area ratio of 1:2.

**Table 1:** Fit parameters used for deconvoluting the XPS spectra for C 1*s* peaks

Chemical identity (binding energy) [76]	Line shape^a^	Binding energy constraint	FWHM constraint
C 1*s sp*^2^ (284.5 eV)	LA (1, 1.6, 50)	None	None
C 1*s sp*^3^ (284.8 eV)	GL (30)	BE (*sp*^2^) + 0.3 eV	None
C 1*s* C–O (285.5–286.5 eV)	GL (30)	BE (*sp*^2^) + 2 eV	Same as *sp*^3^
C 1*s* C=O (287.5–288.9 eV)	GL (30)	BE (*sp*^2^) + 4 eV	Same as *sp*^3^
C 1*s π*–π* (290–292 eV)	GL (30)	290–292 eV	None

^a^GL (30) is a symmetric lineshape that is 30% Lorentzian and 70% Gaussian. LA (1, 1.6, 50) is an asymmetric Lorentzian lineshape numerically convoluted with a Gaussian; at binding energies above the peak maximum, the Lorentzian function is taken to the 1.6 power [77]

FT-IR samples were prepared by mixing approximately 0.2 mg modified GNPs with 300 mg KBr (spectroscopic grade, 99%; Acros Organics) using an agate mortar and pestle then pressed at 10 tons from a hydraulic press for 5 min to obtain a sample disc. Transmission spectra (4000–400 cm^−1^, 32 scans, 4 cm^−1^) were acquired using a Nicolet 5700 FT-IR spectrometer in air. Background spectra were recorded every 10 min.

STORM images were taken using a previously described custom-built STORM system [64], consisting of an Olympus IX-71 inverted fluorescence microscope with Olympus UAPON 100XOTIRFM (NA = 1.49) TIRF oil immersion objective lens in an epi-illumination geometry. Sample movement was controlled using a motorised *x*–*y* stage (PRIOR HLD117) and a PRIOR ProScan III controller. The sample was illuminated by laser beams delivered to the back of the microscope using an optical fibre. A vibration motor was attached to the fibre to remove coherent artefacts in the final image due to laser speckle. Light from the sample was collected by the lens and incident on a Hamamatsu ORCA Flash v2 sCMOS camera. Up to 20000 individual images were acquired for each area with an exposure time of 10 ms. External and internal edges of thiolated CVD graphene were labelled with BODIPY FL L-cystine (Thermo Fisher, *λ*_ex_ = 505 nm, *λ*_em_ = 512 nm, *ε*_max_ = 265 mM^−1^ cm^−1^) and Alexa Fluor 647 maleimide (Thermo Fisher, *λ*_ex_ = 651 nm, *λ*_em_ = 671 nm, *ε*_max_ = 134 mM^−1^ cm^−1^). The graphene samples were immersed in 10 mL deionised water to which 10 mM dye stock in DMSO was added for 2 h at room temperature. After coupling, the samples were washed with water and acetone, dried under a nitrogen stream, and kept at 4 °C and away from light. Image data were processed using ImageJ with the ThunderSTORM plugin [78].

Epifluorescence images were collected on an Olympus BX51 upright microscope using UPlanFLN objectives and captured using a Coolsnap camera (Photometrics) through MetaVue software (Molecular Devices). A specific bandpass filter set for FITC (excitation BP480/40, dichroic Q505LP, emission 535/50) was used.

Scanning electron microscopy (SEM) images were acquired using an FEI Quanta 650 FEG-SEM operating at 1–5 kV. CVD graphene samples on SiO_2_/Si were mounted on 12.5-mm aluminium stubs. No surface coatings were applied.

Specific surface area was determined from the Brunauer–Emmett–Teller (BET) method using a Micromeritics Gemini V surface area and pore size analyser.

Thermogravimetric analysis (TGA) was performed on a TA Instruments Q500 thermogravimetric analyser. 1–3 mg of graphene sample was heated at 10 °C min^−1^ from 30 to 800 °C in a N_2_ atmosphere.

Zeta potential was determined using a Malvern Zetasizer Nano series. Aqueous graphene suspensions (0.05 mg ml^−1^) in deionised water were prepared and placed in disposable foldable capillary cells for testing. The measurement was repeated 6 times for each type of functionalised graphene.

Estimates of the interfacial electron transfer rate (*k*^0^) to pristine and edge-modified CVD graphene samples were carried out in a two-electrode set-up described Valota et al. [79] and Velický et al. [80]. The CVD graphene samples (Graphena) acted as the working electrode when wetted. Graphene was connected to a copper wire (99.9%, 0.15 mm diameter) using a two-part silver-loaded epoxy (RS Components Ltd) cured for 24 h. The connection was coated with a non-conductive epoxy resin (Araldite Rapid) to increase its robustness. An Ag|AgCl quasi-reference electrode was produced by partially exposing Ag in a PTFE-coated wire (99.99%, 0.15 mm diameter) and oxidising it in 0.5 M HCl. The reference was immersed in solution at the top of the pipette and connected to the potentiostat (PGSTAT302N, Metrohm Autolab). A two-electrode set-up, in which the counter and reference electrode are the same, was acceptable because of the small, reversible currents.

Micropipettes were created by pulling borosilicate glass with a micropipette puller (P-87, Sutter Instrument Co.) at 550 °C. The micropipette was filled with 5 mM potassium ferricyanide (99%, Sigma-Aldrich) in 6 M LiCl aqueous solution (99%, Sigma-Aldrich). A high concentration of LiCl was used to prevent droplet evaporation [80]. The filled micropipette was then connected to a pump to control droplet deposition (PV820 Pneumatic PicoPump, WPI) with argon (BOC Industrial Gases, 99.998%).

A camera with a microscope objective (N Plan Apo, Leica Microsystems) and light source were positioned parallel to the sample surface (Figure S7, Supporting Information). Droplet deposition was monitored and recorded using Infinity Analyze software (v.4.2) (Figure S8). A second camera above the sample provided low-resolution, live imaging of the sample to identify areas for deposition. After depositing a droplet, cyclic voltammetry was run using NOVA software (v. 1.11). An uncertainty of 24.4 mV was assigned according to the step size during the scan. Cyclic voltammograms (CVs) were conducted for seven scan rates in nine separate droplets. A set of measurements on a single droplet typically required ca. 30 min, an important consideration in light of the observations by Patel et al. that Fe(CN)_6_^4−^ adsorption on highly oriented pyrolytic graphic (HOPG) lowered electron transfer rates [59]. Typical CVs are shown in Figure S9. The Klingler–Kochi equation was used to calculate *k*^0^ values [81]. A diffusion coefficient of 1.84 × 10^−6^ cm^2^ s^−1^ was used based on previous measurements using the Randles–Ševčík equation [80]. Some electrowetting effects were observed (Figure S10), consistent with others’ reports [82, 83].

## Results and discussion

Graphene/graphite nanoplatelets (GNPs) and monolayer CVD graphene were treated with chlorosulphonic acid and then hydrolysed with base to produce edge-modified graphene sulphonate (G–SO_3_^−^, Scheme 1). The corresponding edge-modified graphene thiol (G–SH) was produced by reduction with triphenyl phosphine and iodine.

Using a combination of analytic techniques was essential to establish not only the identity of the functional groups on the graphene, but also their location on the edges of the graphene.

**Figure 1:**
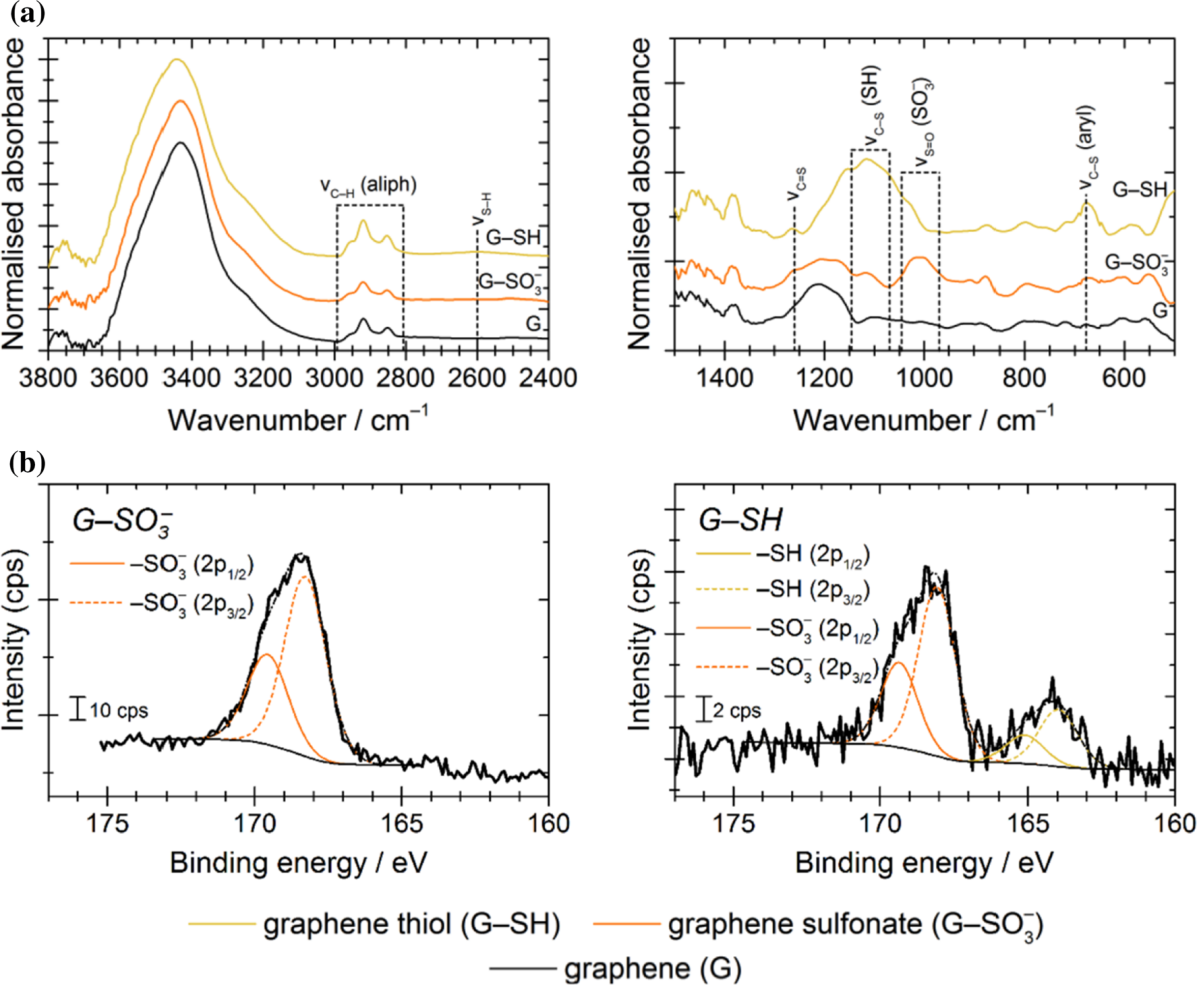
Characterisation of the chemical functionality on edge-modified C750 GNPs by **a** FT-IR and **b** XPS. XPS survey and C 1*s* scans are presented in Figure S3

**Figure 2:**
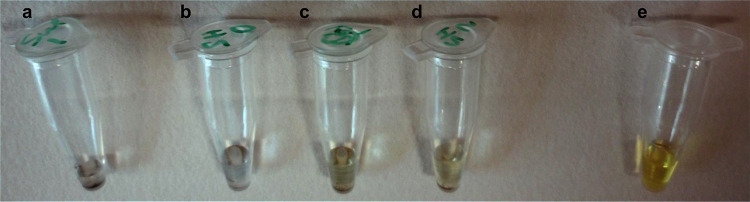
Ellman’s assay for the presence of free sulphhydryl (thiol) groups applied to edge-modified GNPs produced by ultrasonic exfoliation: **a** negative control containing 30 µl of graphene sulphonate and 10 µl Ellman’s reagent solution; **b** negative control containing 30 µl thiographene and 0 µl Ellman’s reagent solution; **c** 30 µl thiographene and 5 µl Ellman’s reagent solution; **d** 30 µl thiographene and 10 µl Ellman’s reagent solution; **e** positive control containing 30 µl 260 mM cysteine and 10 µl Ellman’s reagent solution. [Ellman’s reagent] = 10 mM; [graphene suspensions] = 0.5 g l^−1^

FT-IR analysis (Fig. 1a) was best suited to confirming the presence of functional groups. Sulphur signatures are typically less pronounced in the IR spectrum. Nonetheless, the low-energy region contained the appropriate C–S and S=O vibrations for both the G–SO_3_^−^ and the G–SH, and the G–SH showed a characteristic broad, weak S–H stretch at 2600 cm^−1^. The chemical presence of reactive sulphhydryl groups was further confirmed visually through an Ellman’s assay, in which the reagent becomes yellow coloured in the presence of free thiol groups through a thiol–disulphide exchange reaction. Figure 2 shows that G–SH and a cysteine positive control both produced a colour change, while G–SO_3_^−^ did not.

The XPS of sulphur-modified samples (Fig. 1b) showed the expected signatures for both sulphonate and thiolate groups. Both peaks were clearly asymmetric and could be well described by a deconvolution into S 2*p*_1/2_ and S 2*p*_3/2_ peaks. The sulphonate S 2*p* peaks appeared at higher binding energy (169.5 ± 0.2 eV and 168.1 ± 0.2 eV) than the thiol S 2*p* peaks (165.1 ± 0.2 eV and 164.0 ± 0.2 eV). The S 2*p* spectrum for G–SH showed that the reduction of the sulphonate to the thiol was incomplete (–SO_3_^−^/–SH = 2.8 in the example shown). Quantification of the XPS survey spectra for G–SO_3_^−^ and G–SH gave a S:C atomic ratio of about 0.3:100 (Table 2).

**Table 2:** Physicochemical parameters for the pristine C750 GNPs and edge-modified derivatives^a^

Sample	Raman *I*_*D*_/*I*_*G*_^b^	*L*_*D*_/nm^c^	XPS *sp*^3^:*sp*^2^ atomic ratio^d^	XPS S:O:C atomic ratio^e^	TGA mass change 100–900 °C^f^ (%)	TGA mass loss peak(s) °C^f^
G	0.86	9	0.108 ± 0.011	—:1.2:100	− 10.1	None
G–SO_3_^−^	0.54	11	0.337 ± 0.008	0.33:11.7:100	− 17.4	244 (br), 668
G–SH	0.69	10	0.763 ± 0.118	0.46:7.0:100	− 21.5	221 (br), 356 (br), 447

^a^Additional analyses are given in Table S1, Table S2, Table S5, and Table S6

^b^Values taken from spectra shown in Figure S4

^c^Average distance between defects (Figure S4, Table S5, and Eq. S4)

^d^Taken from the deconvolution of C 1*s* scans shown in Figure S3

^e^Taken from the quantification of survey scans shown in Figure S3. A full analysis is given in Table S1

^f^Traces shown in Figure S17, br = broad

**Figure 3:**
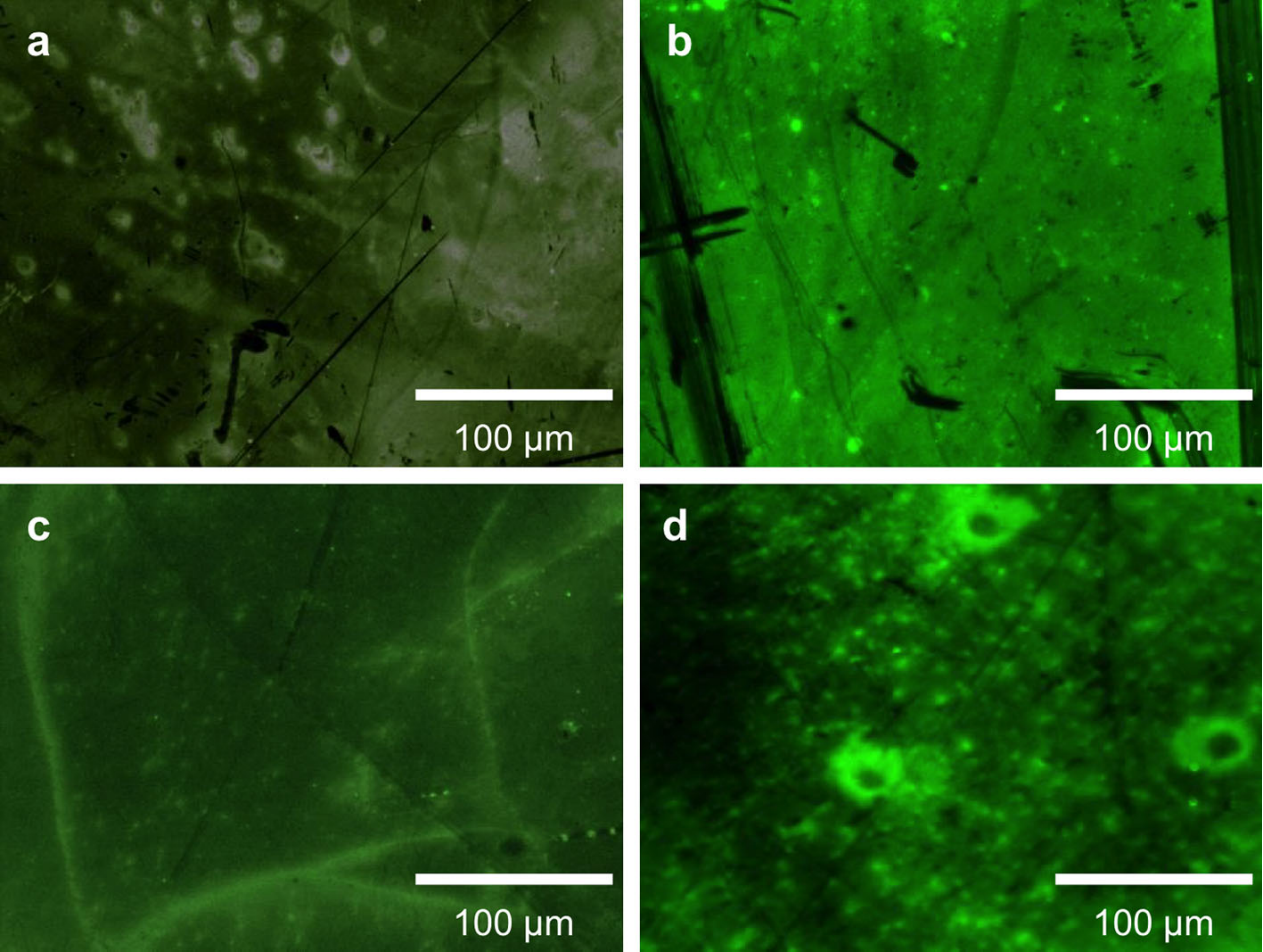
Epifluorescence images of CVD graphene on a silicon wafer with a surface oxide layer: **a** pristine graphene, showing only background fluorescence, **b** pristine graphene incubated with FITC-labelled concanavalin (FITC-ConA) lectin, showing the lectin predominantly adsorbed on the basal surface, **c** mannose-terminated glycographene incubated with FITC-ConA, showing the lectin predominantly bound to the edges, **d** the same sample of glycographene after incubation with excess methylmannoside (an inhibitor of lectin–conjugate binding), displacing the FITC-ConA from the graphene surface

“Glycographene”, an edge-modified graphene bioconjugate, was synthesised through radical addition of allyl mannoside to G–SH (Scheme 1). This bioconjugate was used to highlight the edge specificity of the reactions. Fluorescently labelled ConA lectin (an antinutritional protein that selectively binds to α-D-mannosyl and α-D-glucosyl residues) was used to highlight the edge modification of glycographene. Figure 3 shows that while unmodified graphene adsorbs the lectin non-specifically (Fig. 3b), the lectin binds predominately to the edges of the glycographene (Fig. 3c) and can be displaced by adding excess methylmannoside substrate (Fig. 3d), which binds more strongly than the glycographene. These results are significant because they demonstrate reversible bioconjugation with graphene. This provides a platform for programmable assembly and disassembly of graphene-based nanostructures for regenerative medicine and wound healing. Additionally, surface groups and covalent functionalisation are known to affect carbon nanomaterials’ cell compatibility [28], biodistribution [84], and in vivo degradation [85, 86].

**Figure 4:**
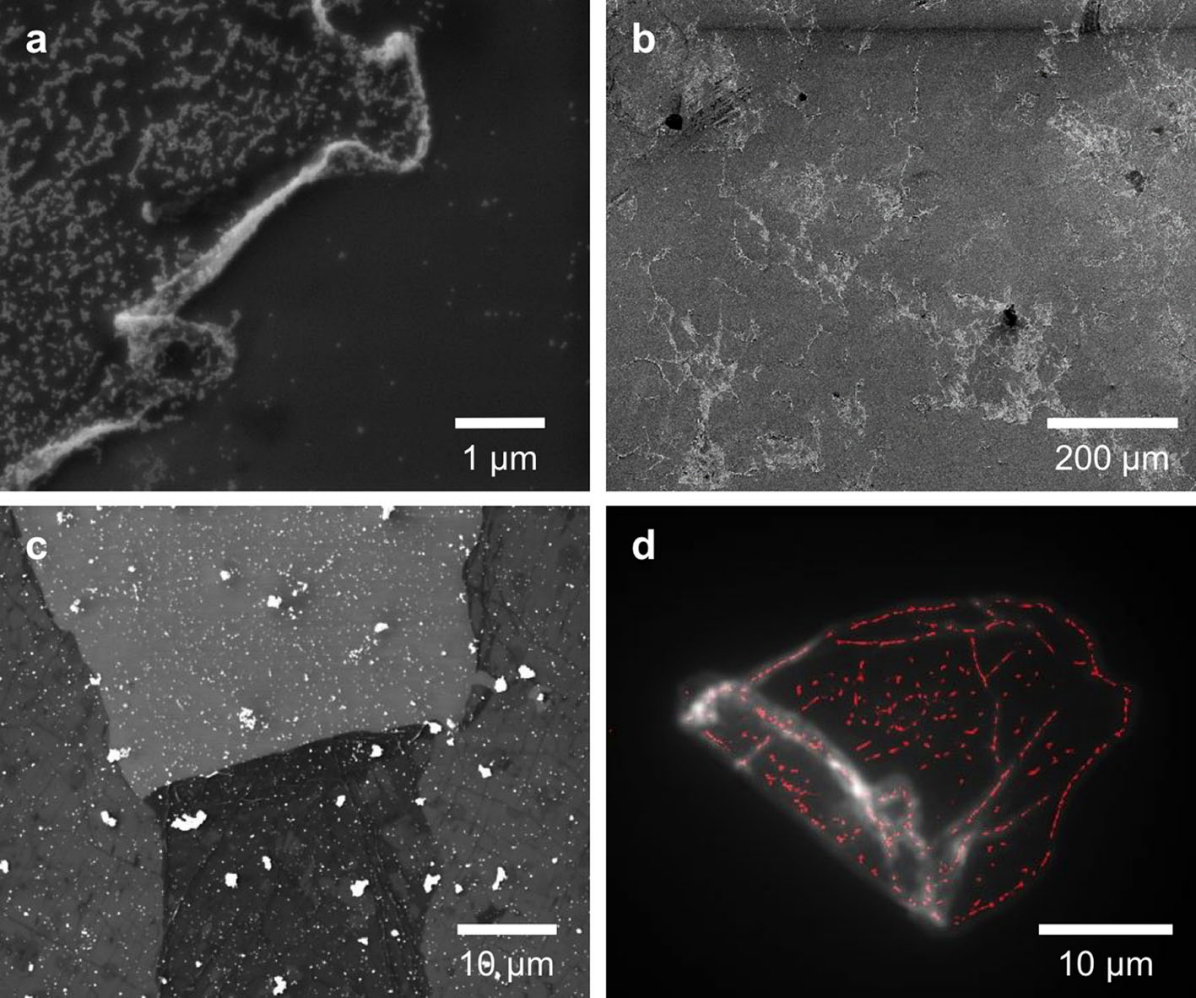
Labelled edge modification sites. **a** SEM image of gold nanoparticles concentrated on the edges of CVD G–SH. The bare SiOx/Si support is visible in the bottom right, **b** wider view SEM image of the same flake of CVD G–SH, **c** SEM image of gold nanoparticles distributed on CVD G–SO_3_^−^ as a negative control. The lighter area in the top middle is the SiOx/Si support. The darker area in the bottom middle is where the torn area of CVD graphene folded over on itself, **d** super-resolution STORM image of CVD G–SH labelled with Alexa Fluor 647 maleimide (red) overlaid with the epifluorescence image of the same flake (greyscale)

Gold nanoparticles were also used to highlight the position of surface thiol groups on CVD G–SH, exploiting the strong Au–S bond. The nanoparticles, which appear as bright circles on the high-magnification SEM image in Fig. 4a, were concentrated at the edges of the G–SH sheets. The basal plane of the graphene also showed some nanoparticle attachment, indicative of thiol modifications at internal edge defects. Figure 4b illustrates the concentration of the nanoparticles at the edges on a larger scale. The negative control using CVD G–SO_3_^−^ (Fig. 4c) showed no concentration of nanoparticles at the edges. The modification of internal edge defects is further illustrated in the super-resolution fluorescence image in Fig. 4d. The maleimido group of the Alexa Fluor probe covalently bonds to thiols. The fluorophore is visible not only along the periphery of the flake, but also in straight-line segments on the interior of the flake, consistent with the presence of line defects where the grains of CVD graphene impinge on each other. Raman maps of another sample of BODIPY-labelled CVD G–SH (Figure S6) showed lower *A*_D_/*A*_G_ values near modified edges than throughout the graphene layer.

The fluorescence from the graphene is initially unexpected. All fluorophores less than 10 nm from graphene or GO are expected to quench their fluorescence due to resonant energy transfer (RET) [87–89], an effect that has been used for contrast enhancement in STORM [66]. This RET is suppressed at edges and other defects in graphene [90]; however, the control experiment in which the dye conjugation procedure was repeated on CVD G–SO_3_^−^ showed no fluorescence. The angle between the dipole in the fluorophore and that in the graphene sheet may be low, greatly reducing the resonant energy transfer [91].

The persistence of *sp*^2^ carbon in the GNPs after edge modification was deduced from deconvolution of the C 1*s* peak (Table 2 and Figure S3), taking advantage of the asymmetry of the *sp*^2^ peak due to the plasmon loss. The C 1*s* signals required an *sp*^3^ component to fit the products of chlorosulphonation and nitration, but there remained a clear asymmetry and *π*–π* shake-up features. These results are consistent with visible light and fluorescence microscopy images of CVD graphene, showing that chlorosulphonation damages the sheets. Some *sp*^2^ character is restored when G–SO_3_^−^ is reduced to G–SH. Few other papers correctly use an asymmetric peak shape to fit the *sp*^2^ character. Both diazonium and Diels–Alder reactions attack the basal plane of graphitic materials [32], so the papers that included a high-resolution C 1*s* XPS plot showed that the spectra of their modified materials had no *π*–π* shake-up peak and generally showed a symmetric C 1*s* peak component at about 285 eV, shifted higher than unmodified graphene or graphite [30, 31, 45–47]. Also, fluorescent imaging of Diels–Alder–modified CVD graphene by Chang et al. [45] showed a uniform fluorescence across their samples, in contrast to the STORM and epifluorescence images shown in the present work. AFM measurements of Diels–Alder–modified CVD graphene also showed a uniform and thick layer consistent with saturated basal plane coverage of the modifier [46].

Raman analysis of the GNPs before and after edge modifications (Table 2 and Figure S4) did not show any consistent trends in the *I*_*D*_/*I*_*G*_ ratio upon modification. There was a lot of variation in this value within single batches of pristine C750 GNPs and their edge-modified analogues, consistent with the Kovtun et al.’s [92] report of wide variation in commercially supplied graphene. The G peaks from the materials produced from GNPs showed a clear *D*′ peak (Figure S4 and Figure S19), caused by a one-phonon defect-assisted electron–defect scattering event from edges, grain boundaries, or internal defects [69]. GNPs produced by ultrasonic exfoliation and their sulphur derivatives had comparable *I*_*D*_/*I*_*G*_ ratios and equally prominent *D*′ features (Figure S19 and Table S5). Larger GNPs (XG C300 and M25) showed a decreasing *I*_*D*_/*I*_*G*_ ratio with increasing GNP size (Figure S23 and Table S7), consistent with the Tuinstra–Koenig relationship [72]. Raman analysis of the GNPs after sulphonation showed no consistent trends in *I*_*D*_/*I*_*G*_ caused by the edge modification. A Raman map of sulphonated CVD graphene (Figure S6) showed that the *I*_*D*_/*I*_*G*_ ratio was *lowered* at the edges, demonstrating that the chemical modifications complicate this simple metric for graphene order. This effect has previously been discussed for graphene with its basal plane modified by diazonium salts [93]. Metrics based on the relative *areas* of the *D* and *G* peaks, namely the average domain diameter, $L_{a}^{\text{A}}$, and average distance between point defects, *L*_*D*_, were more consistent. Sets of modifications to commercial GNPs and graphene produced by mechanically exfoliating graphite in NMP showed little changes in $L_{a}^{\text{A}}$ and *L*_*D*_ on modification relative to the source materials (Table S6). For CVD graphene, *L*_*D*_ consistently dropped by about half and $L_{a}^{\text{A}}$ decreased to about 25–30% of the original value.

Sulphonated graphene (SGnP) has been produced by chemical exfoliation of graphite in chlorosulphonic acid. The work of Abdolmaleki et al. [94] reported a higher amount of sulphur compared to G–SO_3_^−^ in the present study. The higher percentage of sulphur in SGnP indicated to the introduction of a significant number of new defects which could arise from the damage of graphene structure due to the fabrication process. This was evidenced by the Raman spectra, as the *I*_*D*_/*I*_*G*_ ratio was 2.72 for SGnP, in comparison with a low defect (*I*_*D*_/*I*_*G*_ < 1.2) of the edge-modified G–SO_3_^−^ produced in this study, regardless of whether the GNPs or graphite exfoliated in NMP was used (Table S5). The Raman 2D peak shifts to lower energy with decreasing layer number when the number of layers is below ~ 10 [69], but no change in peak shape or shift in 2D peak position was observed for any modification except for C300 GNPs (Figure S4, Figure S19, Figure S23, and Table S7). This observation contrasts with other reports in which chlorosulphonic acid or methanesulphonic acid to exfoliate graphite [94–96].

Raman analysis of CVD graphene and edge-modified derivatives (Table 3 and Figure S5) produced more easily interpretable results. Unmodified CVD graphene had no detectable *D* peak, and a *I*_2*D*_/*I*_*G*_ ratio of over 2 and a FWHM of 24 cm^−1^, indicative of single-layer graphene [70]. The *I*_2*D*_/*I*_*G*_ ratio falls below 1 for bilayer graphene and drops further as the layer number increases. The spectra taken from the edges of the graphene sheet had a measurable but low *I*_*D*_/*I*_*G*_ and lower *I*_2*D*_/*I*_*G*_ ratio, consistent with the literature [97]. Modifications increased the *I*_*D*_/*I*_*G*_ ratio, consistent with the more ragged appearance of the flake by visible light microscopy (Figure S5b, inset) and STORM (Fig. 4d), but the *I*_2*D*_/*I*_*G*_ ratios remained above 2.5, confirming the persistence of single-layer graphene.

**Table 3:** Peak height ratios based on Lorentzian fits to the Raman spectra of unmodified and edge-modified CVD graphene samples shown in Figure S5

Sample	*I*_*D*_/*I*_*G*_	*I*_2*D*_/*I*_*G*_
G (Figure S5a)	0	2.91 ± 0.04
G–S–S–dye (Figure S5a)	0.09 ± 0.01	2.95 ± 0.04
G (centre) (Figure S5b)	0	2.47 ± 0.25
G (edge) (Figure S5b)	0.10 ± 0.01	1.52 ± 0.01
G–SH (Figure S5b)	0.50 ± 0.01	2.72 ± 0.02

Uncertainty values are based on the standard error of the fit and the rules of propagation of uncertainty. Additional analyses (peak areas, *L*_*a*_ and *L*_*D*_) are given in Table S5 and Table S6

Thermogravimetric analyses (TGA) of the samples over a range of 100–900 °C (Table 2 and Figure S17) showed mass losses of between a fifth and a third of the initial mass, as well as distinctive peaks in the derivative traces. The reduced sample, G–SH, showed TGA greater mass loss than starting material, which we attributed to the contribution from strongly adsorbed organic solvent.

**Figure 5:**
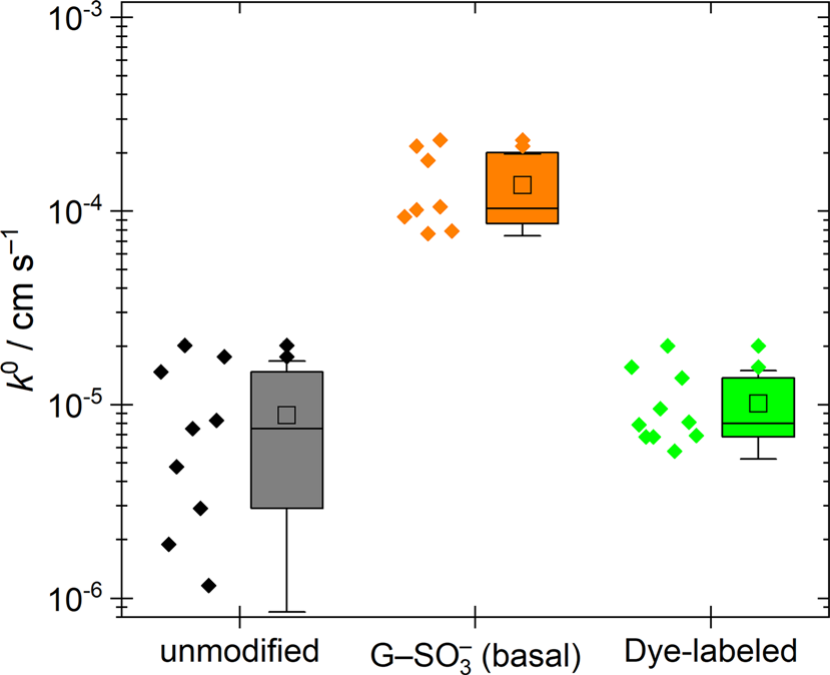
Semilog box-and-whisker representation *k*^0^ values for electron transfer of droplets deposited on the basal plane of unmodified and edge-modified CVD graphene. Data plotted on a linear *y*-axis shown in Figure S13, Figure S14 and Figure S16. Box: 25th, 50th, 75th‰; □ = mean; whiskers = 99% CI

Electrochemical measurements of *k*^0^ on unmodified CVD graphene and CVD G–SO_3_^−^ and dye-labelled G–SH showed no statistically significant difference (*p *= 0.01) between the unmodified samples and the dye-labelled G–SH (Fig. 5). The coefficient for G–SO_3_^−^ was significantly different from the others. The sulphonation may increase the number of defects which are then healed in the subsequent reduction to the thiol or the SO_3_^−^ groups may increase the rate of electron transfer.

Measurements of electron transfer rates to graphite and graphene have been the subject of considerable debate. For example, a ferricyanide probe of electron transfer to the basal plane of HOPG produced *k*^0^ < 10^−9^ cm s^−1^ [98], but later results from Patel et al. [59] showed that the ferricyanide probe can adsorb on the surface and impede electron transfer. The mean value of *k*^0^ for electron transfer to graphene/graphite varied by two orders of magnitude depending on the type of redox probe and measurement conditions [59, 80]. Basal plane kinetics on monolayer graphene have also been shown to be 2–8 times slower than on HOPG [99]. The CVD graphene is not pristine, and there is a contribution to electron transfer from defects. Polymer-free transfer and the use of hydrogenation to control defect density produced a *k*^0^ that stabilised around 1.5 × 10^−4^ cm s^−1^ as defect density increased [100]. Therefore, the CVD graphene used in the present analysis likely had residual surface PMMA.

The *k*^0^ measurements showed consistent relative changes on edge modification. Sulphonation increased the mean *k*^0^ for the basal plane to (1.9 ± 1.5) × 10^−4^ cm s^−1^ (Figure S14 and Figure S15). The large uncertainty in the result is likely due to inhomogeneity of the CVD surface (Figure S6). The mean *k*^0^ for the drops sitting at the edges of CVD G–SO_3_^−^ was (3.6 ± 1.7) × 10^−4^ cm s^−1^ (Figure S14 and Figure S15). Electron transfer rates to the edges of single-layer graphene are expected to be two orders of magnitude greater than the basal plane [98]. The only twofold increase observed here is consistent with the hypothesis that some basal plane droplets will have contributions to their electron transfer rate from internal defects. For dye-labelled CVD G–SH, *k*^0^ reduced to (1.0 ± 0.5) × 10^−5^ cm s^−1^ (Figure S16), comparable to unmodified CVD graphene. Some of the decrease must be attributed to the bulky dye slowing edge electron transfer because the value of *L*_*D*_ estimated from Raman spectra remained about half the value of unmodified CVD graphene, as it was for G–SH (Table S6). The electron transfer rates for the unfunctionalised CVD graphene and dye-labelled CVD graphene are comparable to those of the basal plane of HOPG exposed to air for > 60 min after exfoliation, based on comparable peak separations from scans at 100 mV s^−1^ [58].

The edge modifications produce marked changes in the graphenes’ dispersibility, consistent with the chemical changes (Figure S18 and Table S3). Sulphonation produces graphene that is water dispersible; reduction to the thiol creates a material dispersible only in toluene. Glycographene is moderately dispersible in water, making it a conceivable candidate for applications in biosensors or biomaterials. Contact angle measurements (Table S4) were consistent with these observations.

The edge sulphonation of larger GNPs also produced an unexpected trend. These were presumed to have a smaller amount of edge length for a given mass. Elemental analysis by XPS (Figure S22 and Table S7) showed the opposite trend; however, the largest flakes had the highest S/C ratio. This observation could be an artefact residual, unreacted chlorosulphonic acid trapped between the layers of the GNPs. Consistent with this, graphite has previously been intercalated by tosylate [96] and methanesulphonic acid [95].

The nitration of GNPs (G–NO_2_) was performed and reduced to aminographene (G–NH_2_). FT-IR (Figure S1) confirmed the presence of the nitrogen-containing functional groups. G–NO_2_ showed a distinct, sharp N–O vibration at 1385 cm^−1^ characteristic of nitrated aromatic systems. This signal disappeared when G–NO_2_ was reduced to form G–NH_2_, and distinctive N–H vibrations appeared from 3700 to 3300 cm^−1^ along with C–N stretches and N–H scissoring bands at lower energies.

The N 1*s* XPS spectra could be resolved into three peaks for both G–NO_2_ and G–NH_2_ (Figure S3). The relative amount of the nitro group (*E*_b_ = 406.9 ± 0.3 eV) decreases between the G–NO_2_ sample and its reduced G–NH_2_ analogue. The largest peak at 400.1 ± 0.2 eV can be assigned to organic nitrogen groups, including amines and nitrogen heterocycles [101–105], but tightly adsorbed NMP is likely the main contributor to this peak, consistent with its appearance in almost all samples produced by exfoliating graphite in NMP (Figure S20). This peak’s prominence in both spectra makes it impossible to infer the degree of nitro reduction in the G–NH_2_ sample. A small contribution at 402.8 ± 0.4 eV was attributed to nitrogen in an intermediate oxidation state, such as a hydroxyl amine. Nitration produced products with a greater heteroatom content (N:C around 0.5:100). Analysis of the G–NH_2_ was complicated by the presence of by-products from the nitro reduction over Raney nickel.

As a nitration caused too damaging to CVD graphene, epifluorescence of FITC-labelled GNPs M25 aminographene was an alternative method to highlight the specific edge modification in this study (Figure S2). A faint green fluorescence on the edge of GNP (Figure S2b) could be a possible dye attachment. Nevertheless, conventional fluorescence microscopy was not suitable to observe the edge nitration of GNPs due to a low fluorophore attachment.

## Conclusions

Super-resolution microscopy of fluorophore-labelled edge-modified graphene, the *sp*^3^:*sp*^2^ C ratio, and estimated distance between defects (*L*_*D*_) from Raman are evidence of chemical functionalisation predominantly at the edges and internal defects. This straightforward and scalable method of edge-specific functionalisation creates a versatile platform material for biotechnological and biomedical applications of graphene, rather than graphene oxide or its derivatives. Ongoing and future work on these materials includes measurements of their electronic conductivity and interfacial electron transfer coefficients, applying them in nanoscale theronostics devices, using them as platforms for tissue engineering and regenerative medicine (that is, using the edge group to display specific cues in cell culture), synthesising MRI contrast agents (e.g. with Gd-DOTA–based side groups), creating multiple reactive groups (e.g. both thiol and amine) for bio-orthogonal functionalisation, and assessing whether the mechanical and electrical properties are maintained upon edge functionalisation.

## Supplementary information

Supporting information available: synthesis of nitrographene and aminographene; Raman spectrum analysis methods; representative survey and C 1*s* XPS spectra; elemental analysis based on XPS survey spectra; Raman spectra; schematic for microelectrochemistry and representative measurements; thermogravimetric analysis of pristine and edge-modified GNPs; dispersibility and contact angle measurements; Raman comparison of GNP source for edge sulphonation; Raman and XPS analysis of GNP size on edge sulphonation. In compliance with the University of Manchester’s requirements and those of the funding agencies, these data for this work are available to download from https://data.mendeley.com/datasets/mwrvrcj639/draft?a=20782f0e-4a57-4056-a4e7-c24057beded6

## Electronic supplementary material

Below is the link to the electronic supplementary material.

(PDF 3805 kb)

## Data Availability

In compliance with the University of Manchester’s requirements and those of bodies that funded this work, the data associated with this work are available to download from 10.17632/mwrvrcj639.1.
