# Supplementary material for: A versatile route to edge-specific modifications to pristine graphene by electrophilic aromatic substitution
Source: J Mater Sci. 2020 May 9;55(24):10284–302. doi: 10.1007/s10853-020-04662-y (PMC7266800; doi:10.1007/s10853-020-04662-y)
Supplement: Supplementary file 1 — (PDF 3805 kb) [file 10853_2020_4662_MOESM1_ESM.pdf]

Supporting information (SI)

A versatile route to edge-specific modification of pristine graphene by electrophilic aromatic substitution

Philippa M. Shellard, Thunyaporn Srisubin, Mirja Hartmann, Joseph Butcher, Fan Fei, Henry Cox, Ashley M. Shepherd, Thomas P. McNamara, Trevor McArdle, Robert M.J. Jacobs, Thomas A. Waigh, Sabine L. Flitsch and Christopher F. Blanford

Table of Contents

S1. Synthesis of nitrographene and aminographene 2

S2. Raman spectrum analysis 4

S2.1 Fitting method 4

S2.2 Equations used to estimate graphene domain size and defect distance 4

S3. FTIR, XPS and STORM analysis of nitrographene and aminographene 7

S4. XPS spectra and elemental analysis 8

S5. Raman spectra 10

S6. Microelectrochemical measurements to determine k^0 12

S7. Thermogravimetric analysis 16

S8. Dispersibility and contact angle measurements 17

S9. Effect of GNP source 19

S10. Effects of GNP size 24

S11. Zeta potential measurements 26

S11. References 27

S1. Synthesis of nitrographene and aminographene

Nitrographene (G–NO_2, **Scheme S1**) was synthesised by adding 100 mg GNPs to an ice-cold mixture of 20 mL 70 % nitric acid and 28 mL concentrated sulfuric acid. The reaction mixture was stirred and left at room temperature for 21 h. The mixture was poured over ice and neutralised in 5 M aqueous NaOH solution to pH 7. The solids were isolated by vacuum filtration through a 0.45 µm HV membrane filter (EMD Millipore), washed with deionised water and freeze-dried.

Aminographene (G–NH_2, **Scheme S1**) was synthesised by decanting a mixture of 50 mg of nitrographene and 3 ml of aqueous Raney nickel suspension into 40 mL methanol at room temperature. Then, 0.76 g of sodium borohydride was slowly added intro the mixture, keeping temperature between 30°C and 40°C.[1] The reaction stirred at room temperature for 24 h, vacuum filtered through a 0.45 µm HV membrane, washed with deionised water and freeze-dried.

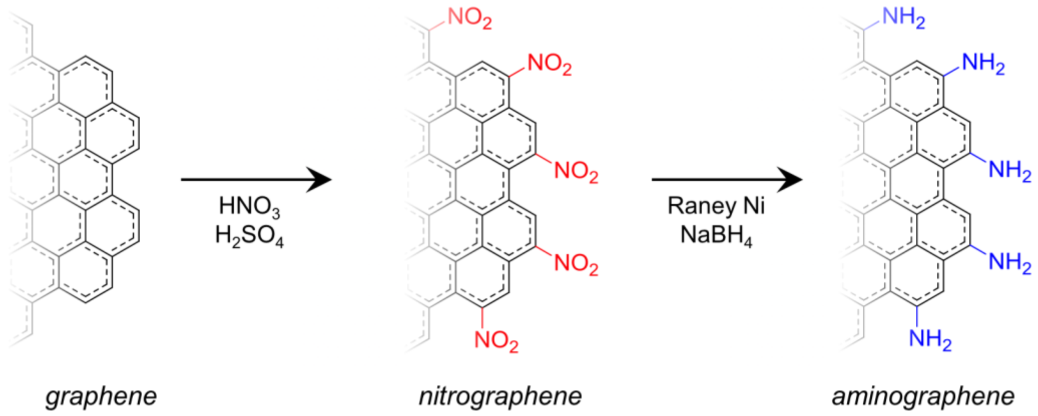

Scheme S1. Synthesis of nitrographene (G–NO_2) by electrophilic aromatic substitution, and its subsequent reduction to aminographene (G–NH_2).

S2. Raman spectrum analysis

S2.1 Fitting method

Raman spectra were fit in Origin based on the methods put forward by Puech and co-workers.[2] The D peak was fit to a double Voigt model (i.e., two Voigt peaks sharing a single centre point). The G and D′ peaks were fit to Voigt peak shapes. The separation between the G and D peaks was sufficient that no asymmetry needed to be included in the G peak shape.

S2.2 Equations used to estimate graphene domain size and defect distance

Puech and co-workers also provided a useful summary of equations used to estimate the domain size (L_a) in graphenic materials as well as the distance between point defects.[2]

The classic equation from Tuinstra and Koenig [3] predicts the domain size based on the relative *intensity* of the D band:

$$L_a^{TK} = \frac{4.4\ \text{nm}}{I_D/I_G} \tag{S1}$$

The more recent work from Cançado et al.[4] uses the relative *area* of the D band and accounts for the energy of the Raman excitation source (E_L):

$$L_a^{A} = \frac{560\ \text{nm eV}^4}{E_L^4}\frac{1}{A_D/A_G} \tag{S2}$$

The D peak's linewidth (the halfwidth at half maximum, HWHM_D) also provides an estimate of the domain size:[5]

$$L_a^{HWHM} \cong \frac{300\ \text{nm cm}^{-1}}{\text{HWHM}_D} \tag{S3}$$

The average spacing between point defects (L_D) can be estimated in a similar way to L_a^A:[6]

$$L_D = \sqrt{\frac{3640\ \text{nm}^2\ \text{eV}^4}{E_L^4}\frac{1}{A_D/A_G}} \tag{S4}$$

S3. FTIR, XPS and STORM analysis of nitrographene and aminographene

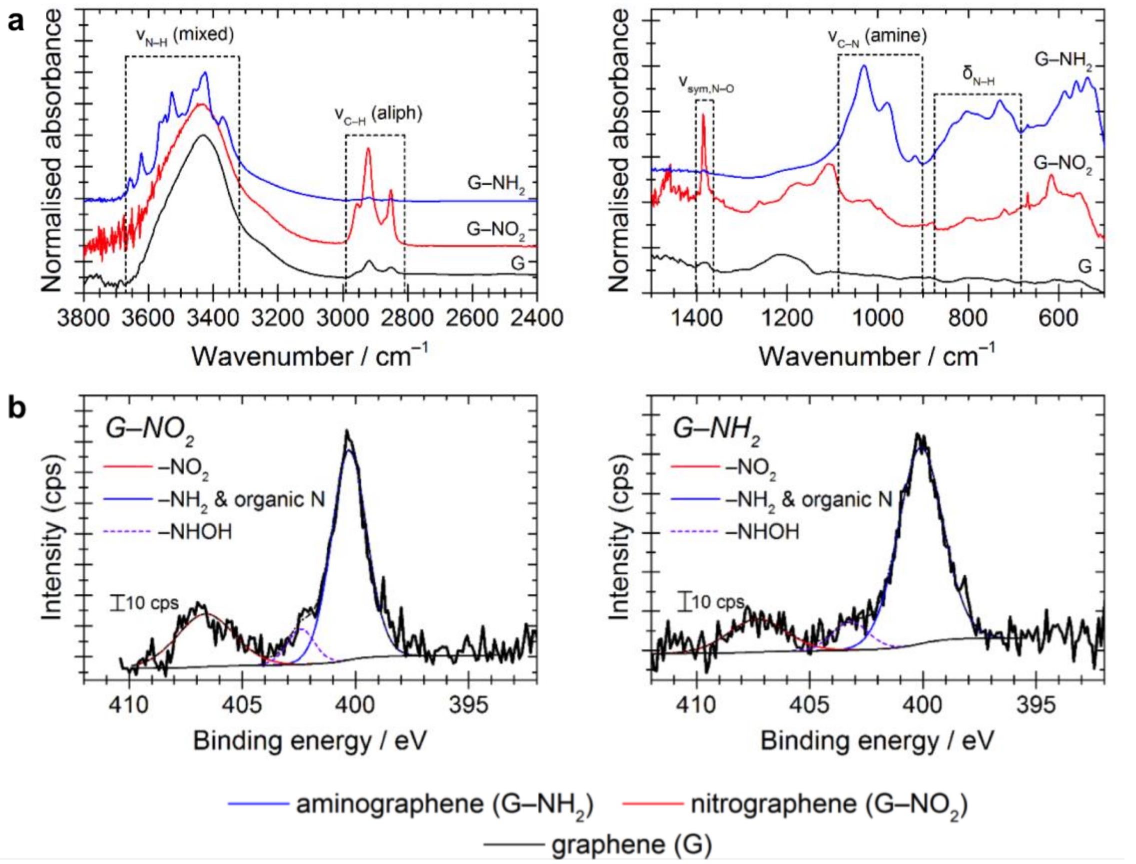

Figure S1. Characterisation of the chemical functionality on G–NO_2 and G–NH_2 from C750 GNPs by (a) FT-IR and (b) XPS. XPS survey and C 1s scans are presented in **Figure S3**.

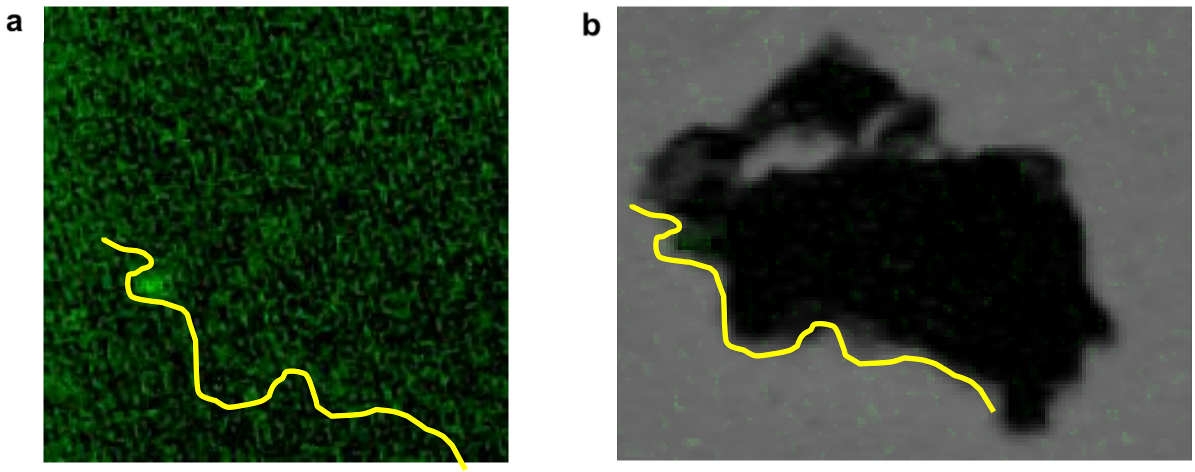

Figure S2. Epifluorescence images of M25 aminographene labelled with FITC: (a) green fluorescence image, (b) a composite of green fluorescence image, with the corresponding visible light image of a GNP.

S4. XPS spectra and elemental analysis

Table S1. Elemental concentrations (in at%) of pristine GNPs and their edge-modified analogues determined by XPS survey spectra (**Figure S3** and **Figure S22a**).

Sample (source)	C	N	O	S	O:C	N:C	S:C	Others
G (C750)	98.73±0.68	—	1.17±0.07	—	0.012±0.001	—	—	Si: 0.10±0.04
G (NMP)	86.19±0.49	—	4.81±0.26	—	0.056±0.003	—	—	F: 7.10±0.20<br>Al: 0.94±0.31<br>Si: 0.97±0.30
G–SO_3^- (C750)	89.01±0.09	0.09±0.07	10.39±0.06	0.29±0.02	0.117±0.001	0.0010±0.0008	0.0033±0.0003	Na: 0.11±0.01<br>Cl: 0.11±0.01
G–SO_3^- (NMP)[a]	22.06±0.45	0.53±0.18	49.09±0.45<br>(20.86±0.73)	2.30±0.21	2.226±0.050<br>(0.946±0.038)	0.024±0.008	0.104±0.010	Al: 18.82±0.46<br>Si: 7.20±0.40
G–SO_3^- (C300)	95.29±0.09	0.15±0.06	3.83±0.05	0.14±0.02	0.040±0.001	0.0016±0.0006	0.0037±0.0003	Na: 0.11±0.01<br>Si: 0.35±0.03<br>Cl: 0.13±0.01
G–SO_3^- (M25)	89.01±0.06	0.09±0.07	10.39±0.06	0.29±0.02	0.177±0.02	0.0295±0.0018	0.0016±0.0004	Cl: 0.50±0.04<br>Na: 0.21±0.02
G–SH (C750)[a]	92.58±0.37	0.47±0.22	6.46±0.27	0.43±0.18	0.070±0.003	0.0051±0.0026	0.0046±0.0125	Cl: 0.05±0.04
G–SH (NMP)	60.08±0.78	1.31±0.41	25.17±0.50<br>(13.89±0.86)	1.81±0.28	0.419±0.010<br>(0.231±0.015)	0.022±0.005	0.030±0.047	Na: 0.25±0.11<br>Al: 7.52±0.57<br>Si: 3.84±0.44
G–NO_2 (C750)	77.91±0.12	0.26±0.07	15.38±0.08	—	0.197±0.001	0.0033±0.0008	—	Si: 6.45±0.09
G–NO_2 (NMP)[a]	84.80±0.61	0.28±0.37	9.81±0.29<br>(5.58±0.59)	—	0.117±0.004<br>(0.066±0.007)	0.0033±0.0044	—	Na: 0.44±0.08<br>Al: 2.82±0.42<br>Si: 1.85±0.24
G–NH_2 (C750)[a]	29.99±0.25	0.36±0.06	42.23±0.21<br>(16.03±0.29)	—	1.408±0.016<br>(0.535±0.012)	0.0019±0.0020	—	Na: 0.87±0.04<br>Ni: 2.76±0.09<br>Al: 17.46±0.25<br>Si 6.34±0.13
G–NH_2 (NMP)	85.67±0.69	0.11±0.51	11.36±0.43	—	0.132±0.005	0.0013±0.00	—	Na: 0.55±0.14<br>2.31±0.34

[a] Values in brackets remove the oxygen content assuming the aluminium is present as Al_2O_3.

Table S2. Estimates of non-graphenic carbon from XPS sp^3:sp^2 ratios in addition to those given in **Table 2**. Source spectra shown in **Figure S3** and **Figure S20**.

Sample	Source[a]	XPS sp^3:sp^2 atomic ratio
G	NMP	1.223 ± 0.708
G–SO_3^-	NMP	—[b]
G–SH	NMP	1.682 ± 1.051
G–NO_2	NMP	1.314 ± 0.595
G–NH_2	NMP	0.509 ± 0.277
G–NO_2	C750	1.083 ± 0.092
G–NH_2	C750	9.482 ± 9.457

[a] NMP = graphite sonochemically exfoliated in *N*-methyl-2-pyrrolidone, C750, C300, M25 = GNP from XG Sciences. [b] No sp^2 carbon detected; (6.83 ± 2.29) at% of C 1s assigned as C–SO_3.

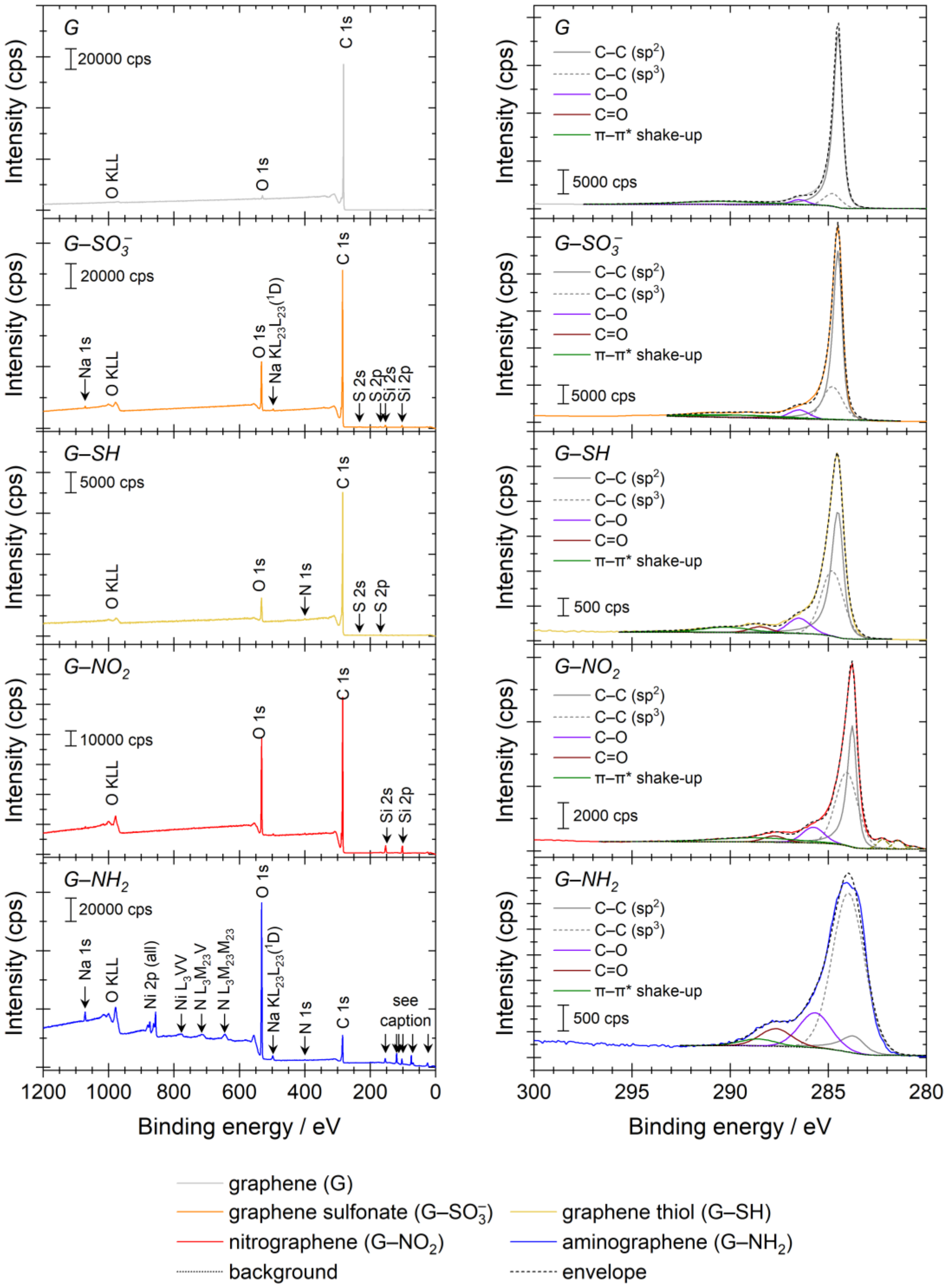

Figure S3. XPS survey scans (left column) and C 1s high-resolution scans (right column) of C750 GNPs and their edge-modified analogues. Low binding energy peaks in G–NH$_2$ survey (L–R): Si 2s (154 eV), Al 2s (119 eV with Ni 3s shoulder), Si 2p (103 eV), Al 2p (74 eV), Ni 3p (68 eV), O 2s (25 eV).

S5. Raman spectra

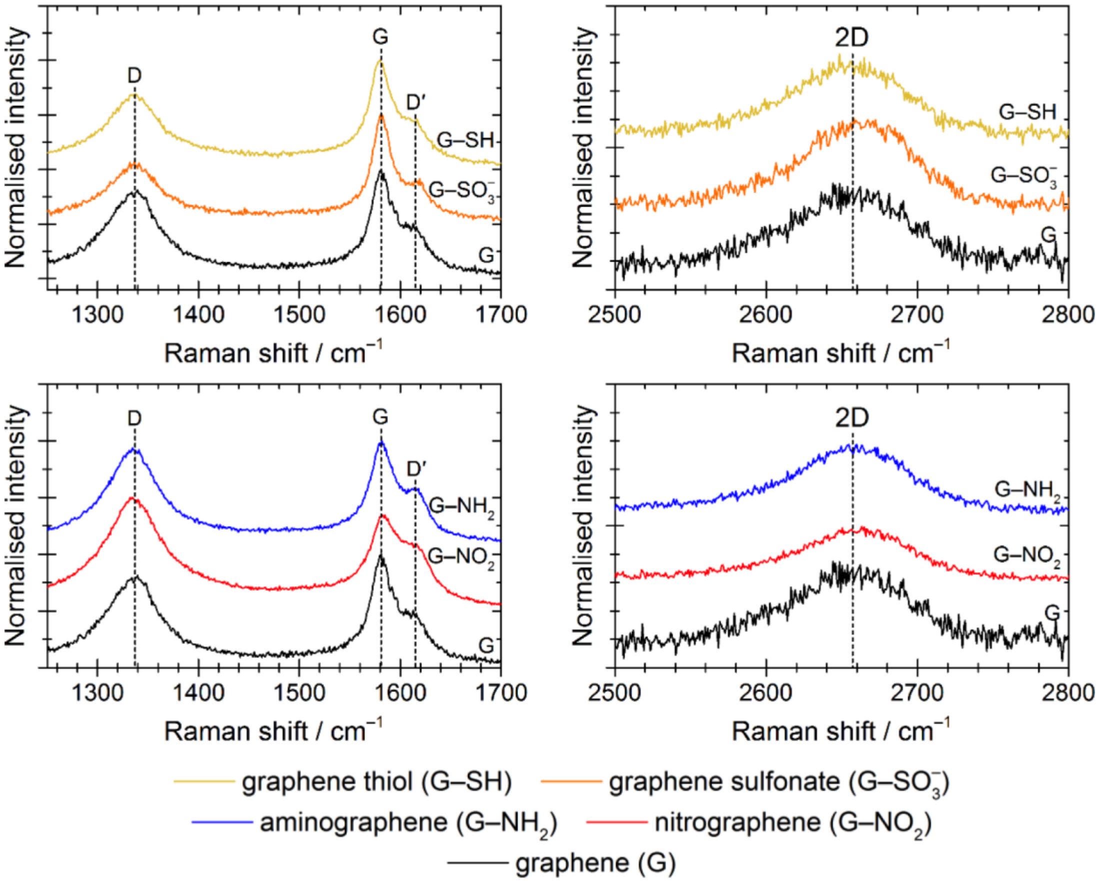

Figure S4. Raman spectra of edge-modified C750 GNPs relative to the unmodified GNPs (λ=633 nm). Intensity is normalised to G peak intensity.

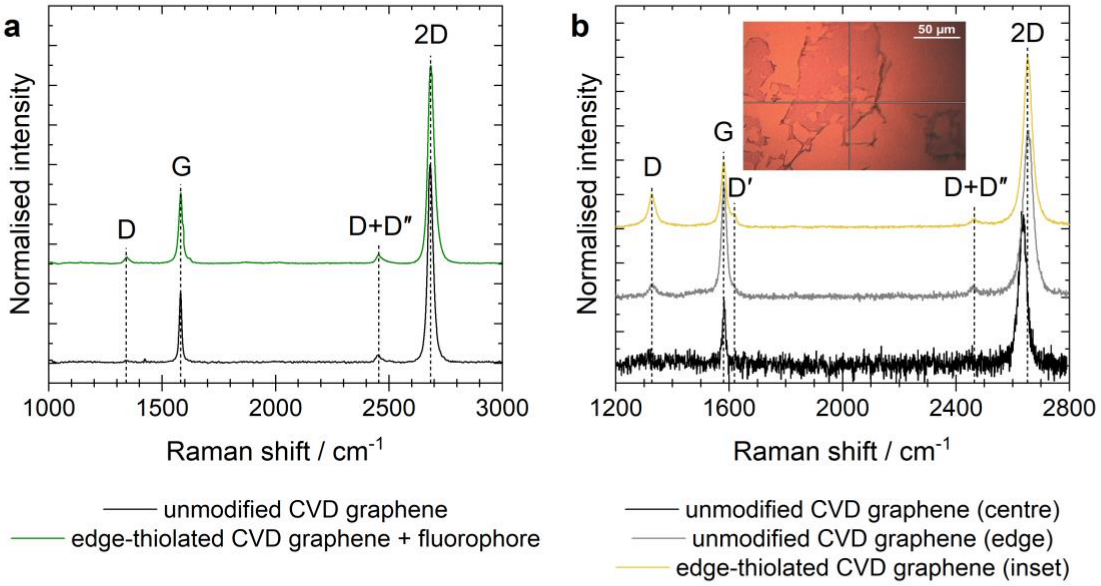

Figure S5. Raman spectra (λ=633 nm) showing changes in relative peak intensities caused by edge-modifications of CVD graphene: (a) BODIPY FL L-cysteine attached to CVD G–SH, (b) G–SH compared to the edge and centre of an unmodified sheet of CVD graphene. Inset shows a visible-light image of the spot from which the G–SH spectrum was recorded. Intensity is normalised to 2D peak intensity. The area probed by the Raman analysis of the edge-thiolated sample in panel (b) included some edges, leading to a higher I_D/I_G ratio of than the dye-labelled graphene in panel (a).

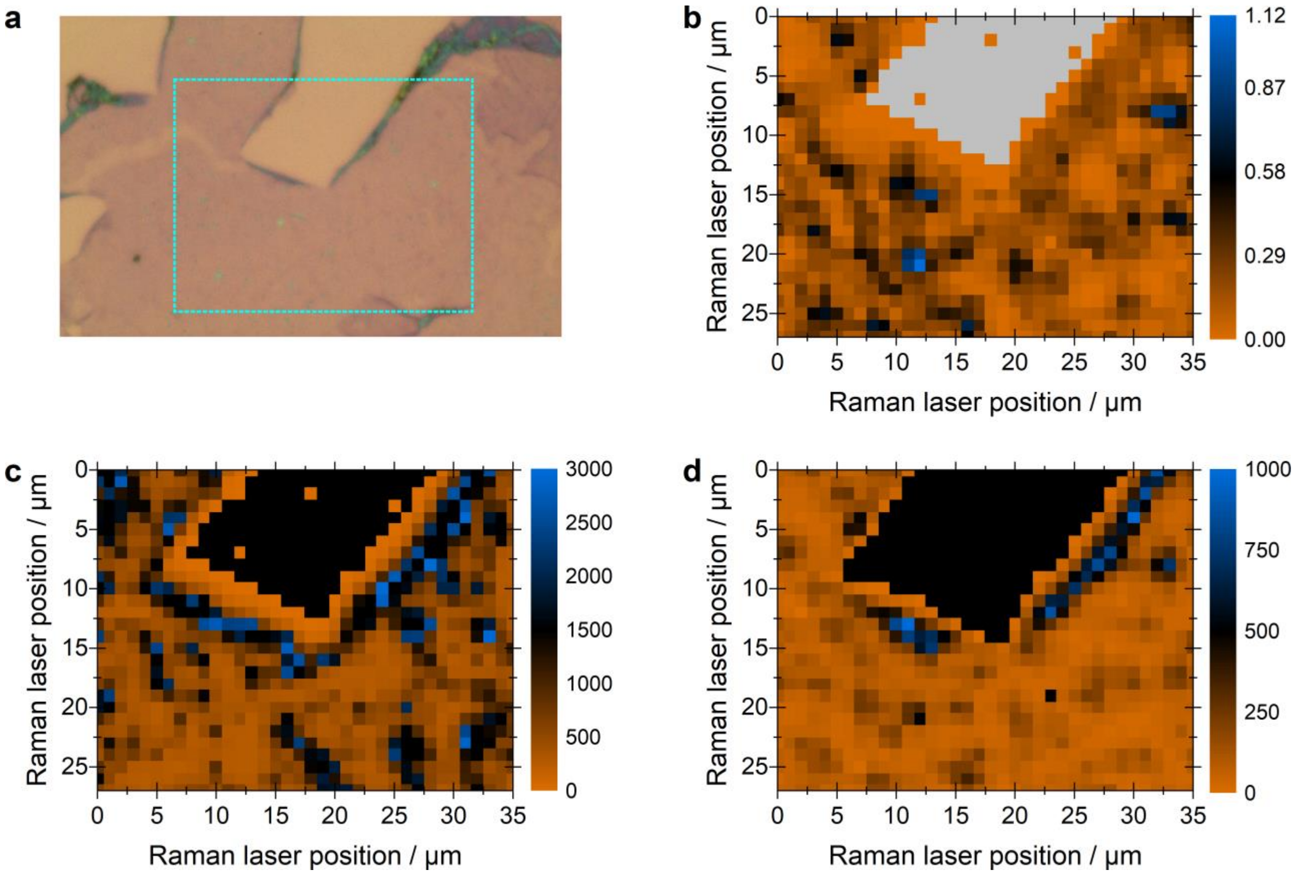

Figure S6. Raman maps of CVD G–SH coupled to BODIPY–FL L-cysteine:(a) visible light reflection image, dashed cyan box indicates scanning area, lighter areas in centre-top and top-left are areas without CVD graphene (i.e., bare SiO_2 on Si); (b) A_D/A_G map based on data shown in panels (c) & (d), areas where A_D or A_G were negative are grey; (c) integrated G peak intensity minus linear baseline background correction, negative values appear black; (d) integrated D peak intensity minus linear baseline background correction, negative values appear black.

S6. Microelectrochemical measurements to determine k_0

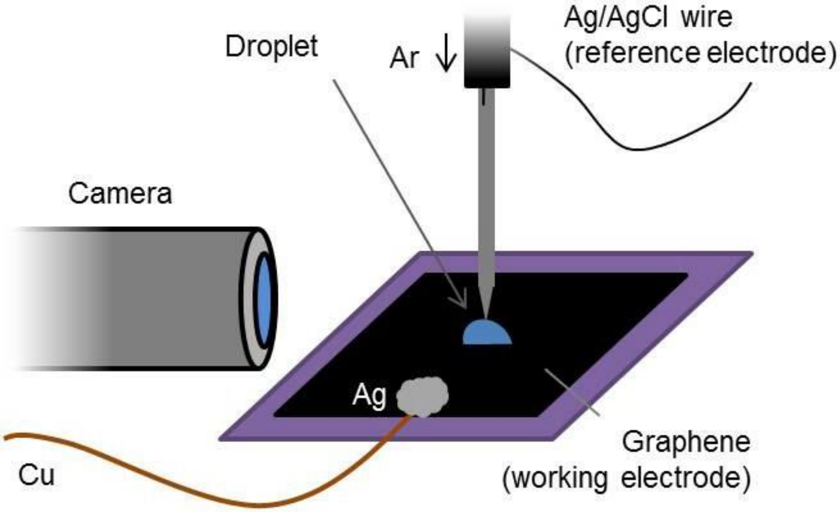

Figure S7. Schematic of the microelectrochemistry apparatus.

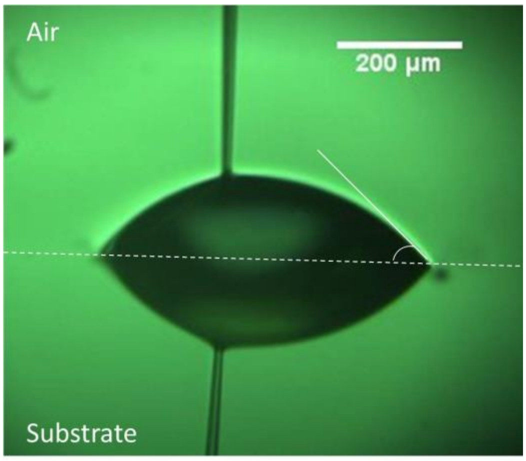

Figure S8. A typical droplet after deposition on non-functionalised CVD graphene. The camera is focused parallel to the plane of the sample, as seen in figure 4.2. The substrate is reflective resulting in a mirror image of the droplet and pipette appearing underneath the real image. The relatively small contact angle may be due to oxygen-containing deposits.[7]

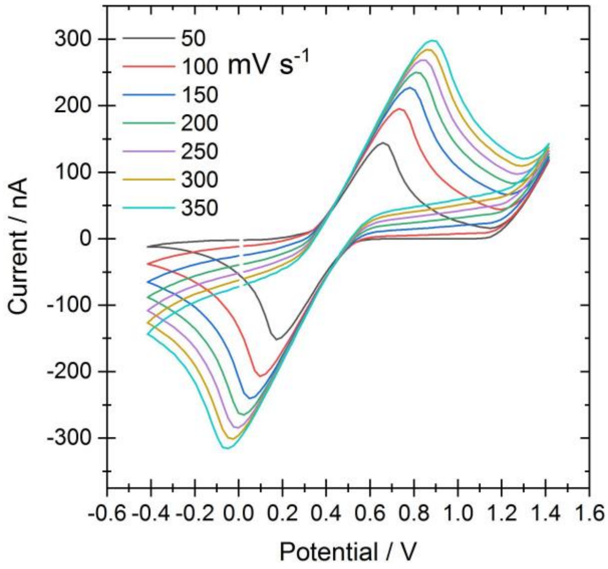

Figure S9. Cyclic voltammogram traces associated with the droplet in **Figure S8**. Conditions: 5 mM potassium ferricyanide in 6 M LiCl; temperature: 20 °C; potential versus Ag|AgCl QRE.

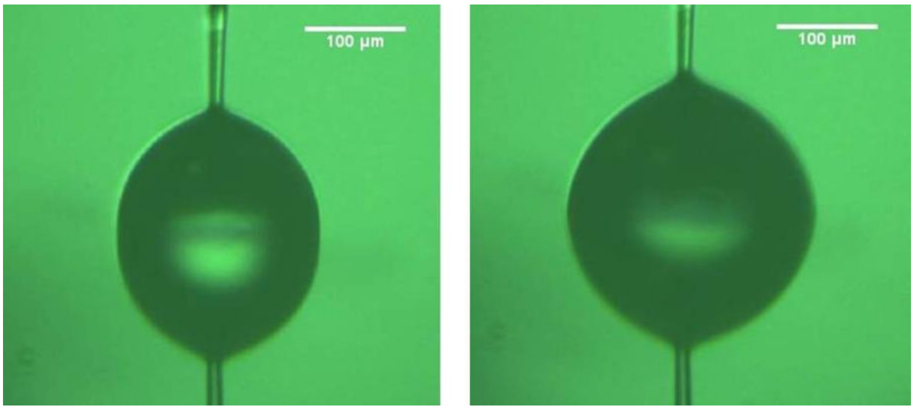

Figure S10. Deposited droplets of 5 mM potassium ferricyanide in 6 M LiCl. Left: before potential cycling. Right: during cycling. Increase in potential results in ’spreading’ and a reduction in contact angle. The contact angle becomes fixed after to this new configuration.

Peak potentials differences (ΔE_p) were measured for each scan rate and droplet. Seven ΔE_presults were taken from varying the scan rate for each droplet. The resulting k^0 values are shown in **Figure S11**.

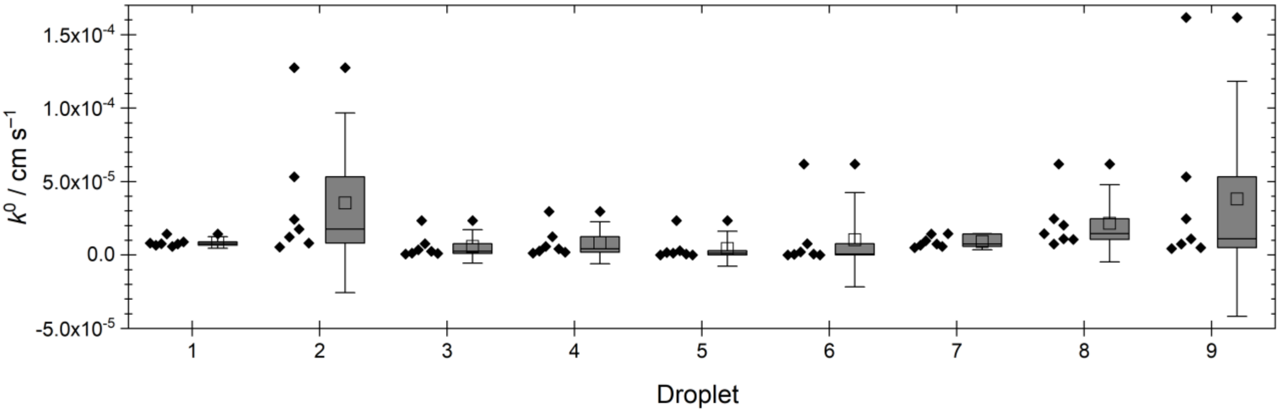

Figure S11. Box-and-whisker chart of the k^0 values on non-functionalised CVD graphene. Data points to the left of the boxes are for each scan rate including 50 mV s^{-1}. Box: 25th, 50th, 75th percentile; ☐ = mean; whiskers = 99% CI.

There was a high apparent outlier in 8 of 9 droplets. Some scans recorded at 50 mV s^{-1} showed a smaller ΔE_p, but k^0 should be the same for all scan rates.[8] Therefore, the mean k^0 for each scan rate was calculated (**Figure S12**). A Grubb’s test for outliers was run ($\alpha = 0.05$) and the 50 mV s^{-1} results were disregarded (**Figure S13**). The revised mean k^0 was $(8.8 \pm 7.1) \times 10^{-6}$ cm s^{-1}.

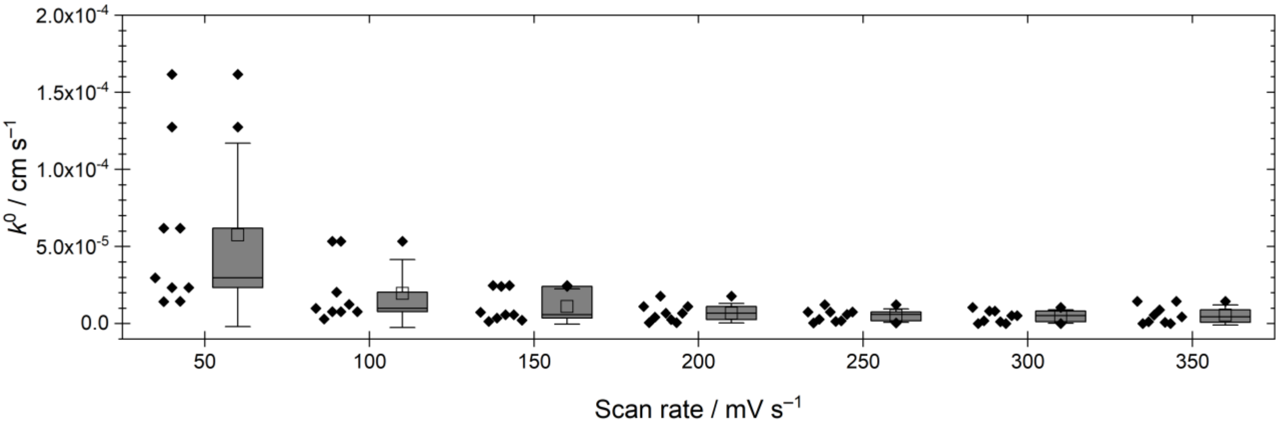

Figure S12. Box-and-whisker chart of the k^0 values on non-functionalised CVD graphene, grouped according to scan rate. Box: 25th, 50th, 75th percentile; □ = mean; whiskers = 99% CI.

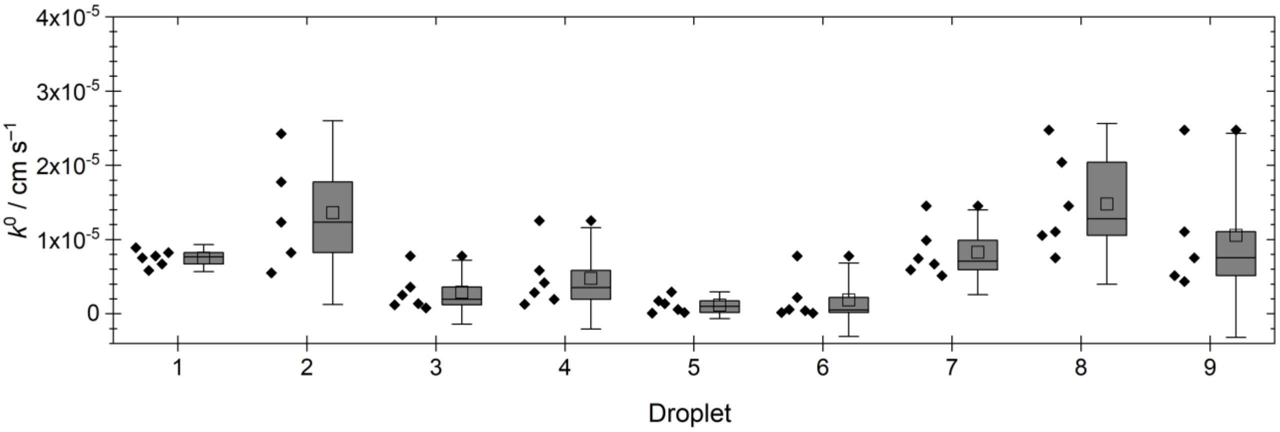

Figure S13. Box-and-whisker chart of k^0 values corresponding to those in **Figure S11** after Grubb's test for outliers, values for 50 mV s^{-1} removed. Box: 25th, 50th, 75th percentile; □ = mean; whiskers = 99% CI.

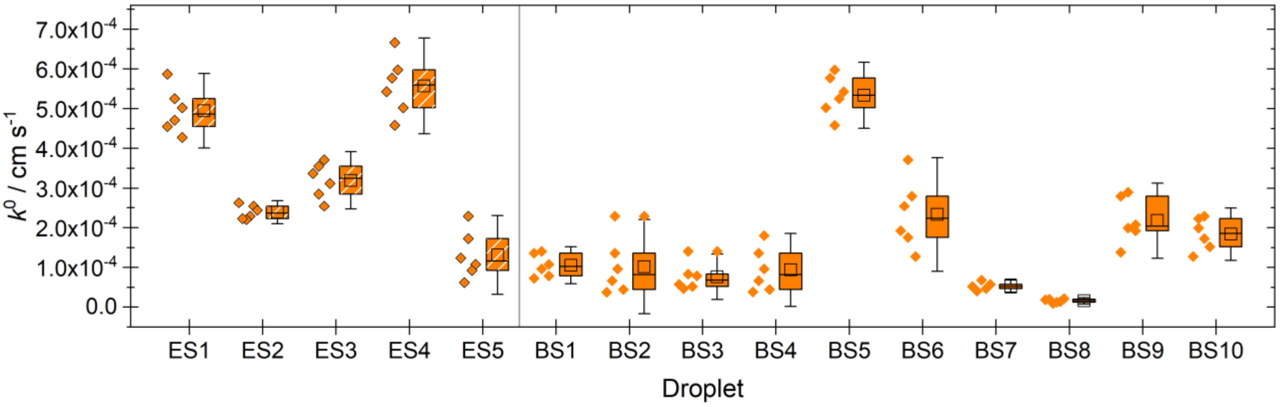

Figure S14. Comparative box-and-whisker chart of the k^0 values on the basal plane and edge of CVD G–SO_3^-. Box: 25th, 50th, 75th percentile; □ = mean; whiskers = 99% CI.

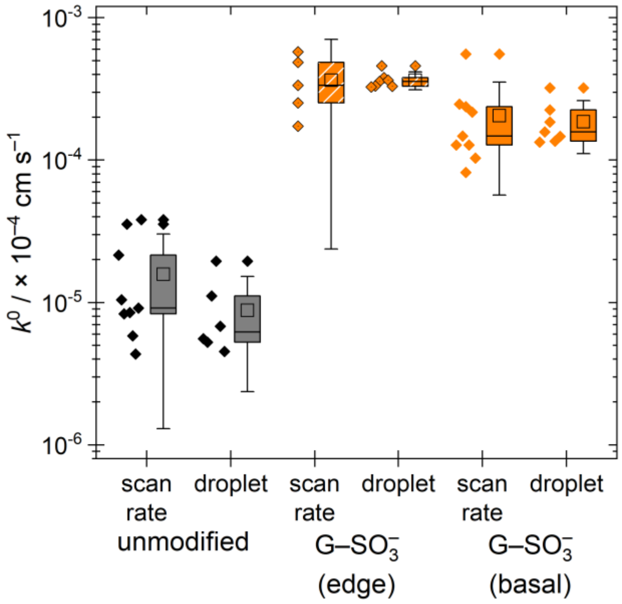

Figure S15. Semilog box-and-whisker chart comparing the k^0 values unmodified and sulfonated CVD graphene. Data plotted on a linear y-axis shown in **Figure S13** and **Figure S14**. Box: 25th, 50th, 75th percentile; ☐ = mean; whiskers = 99% CI.

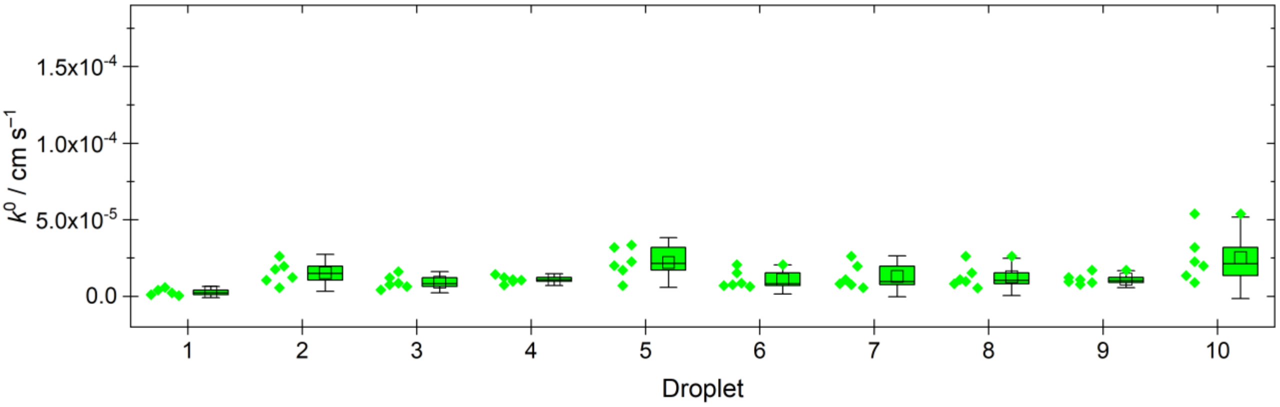

Figure S16. Box-and-whisker chart of the k^0 values on the basal plane of dye-labelled CVD G–SH. Box: 25th, 50th, 75th percentile; ☐ = mean; whiskers = 99% CI.

S7. Thermogravimetric analysis

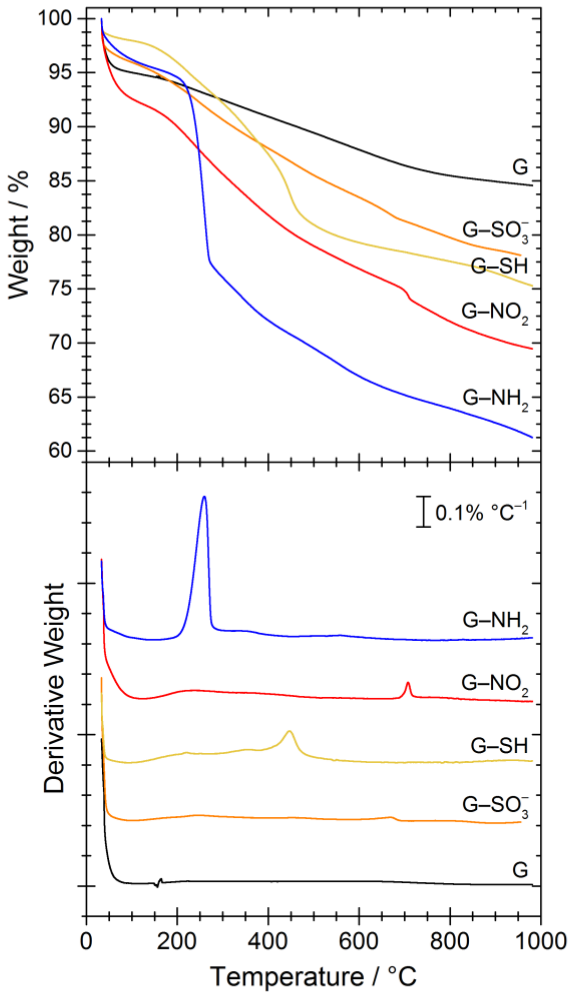

Figure S17. Representative thermogravimetric analysis traces from C750 GNPs and its edge-modified analogues recorded under N_2 atmosphere.

S8. Dispersibility and contact angle measurements

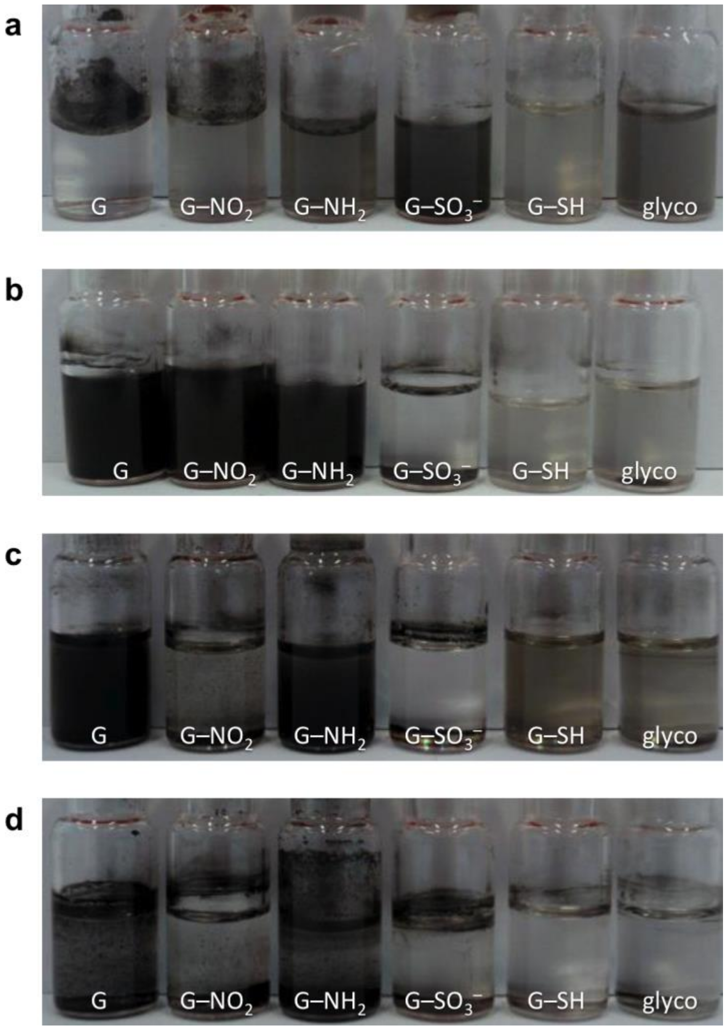

Figure S18. Photographs illustrating the tuneable dispersibility of graphene produced by ultrasonic exfoliation in: (a) water, (b) ethanol, (c) toluene, (d) cyclohexane. Graphene type (left to right): graphene produced by ultrasonic exfoliation with no functionalisation, nitrographene (G–NO_2), aminographene (G–NH_2), graphene sulfonate (G–SO_3^-), graphene thiol (G–SH), glycographene (terminated with α-D-mannose). The white balance of the images was adjusted by setting the shadow to a neutral grey. Concentration: 1 g l^{-1}.

Table S3. Qualitative comparison of dispersibility of edge-modified graphene produced by ultrasonic exfoliation of graphite in NMP.

Material	Water	Ethanol	Toluene	Cyclohexane
Graphene (ultrasonic exfoliation)	Poor	Good	Good	Good
Graphene sulfonate	Good	Poor	Poor	Poor
Graphene thiol	Poor	Poor	Moderate	Poor
Nitrographene	Moderate	Good	Moderate	Poor
Aminographene	Moderate	Good	Good	Poor
Glycographene (α-D-mannose)	Moderate	Poor	Moderate	Poor

Table S4. Contact angle measurements of water on Anodisc filter coated with a laminate of edge-modified GNPs produced by ultrasonic exfoliation.

Surface	Contact angle
Bare Anodisc	26°
Unmodified GNPs	105.1 ± 7.67°
Graphene sulfonate ($G–SO_3^-$)	45.9 ± 5.7°
Graphene thiol (G–SH)	66.2 ± 3.6°
Nitrographene ($G–NO_2$)	75.2 ± 4.5°
Aminographene ($G–NH_2$)	not observed

S9. Effect of GNP source

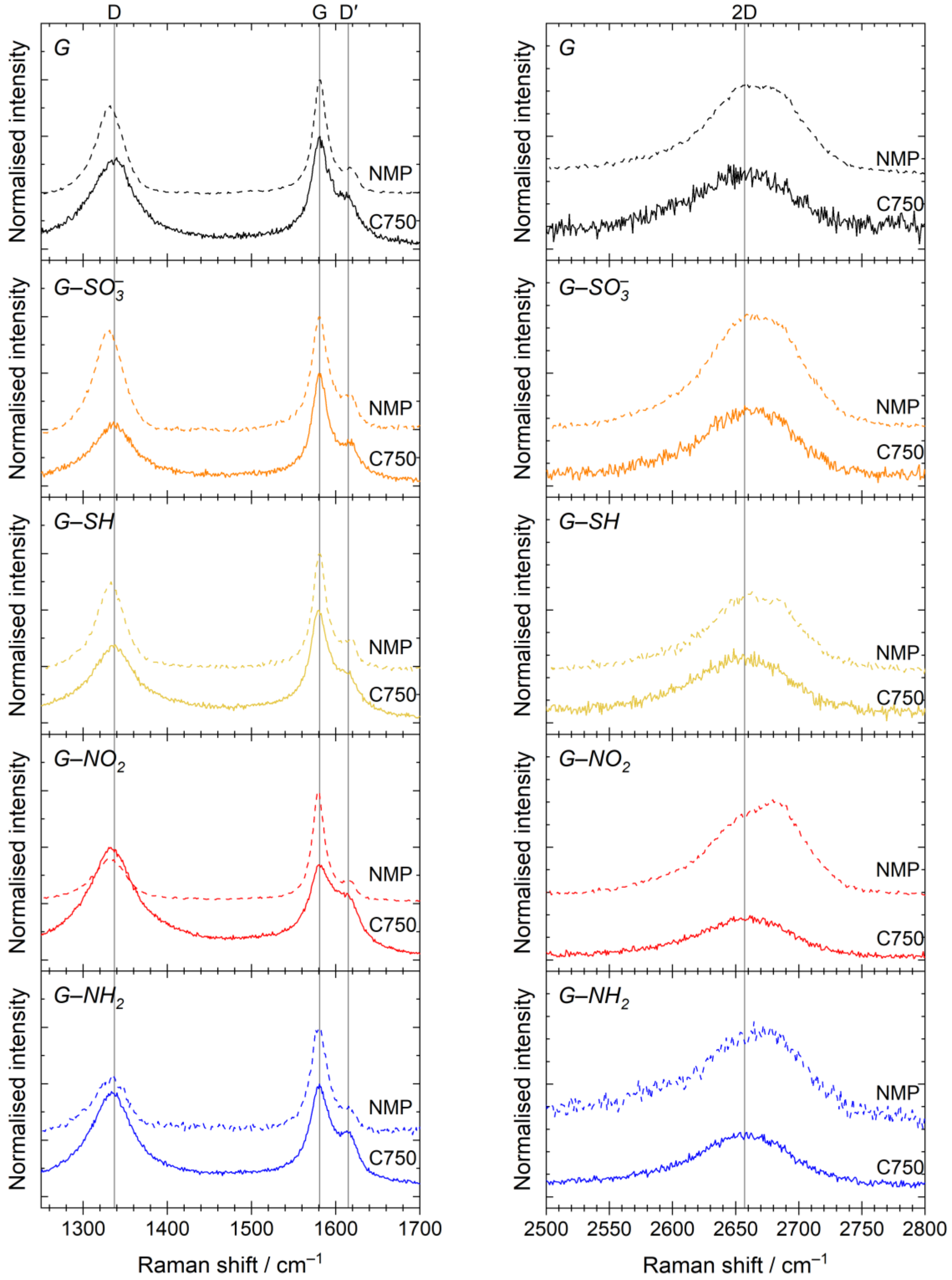

Figure S19. Comparison of the Raman spectra of GNPs produced by ultrasonic exfoliation in NMP versus commercially produced C750 GNPs. λ=633 nm (E_L = 1.96 eV) for NMP samples and λ=514 nm (E_L = 2.14 eV) for C750 samples.

Table S5. Summary of Raman disorder metrics for the GNPs and their sulfur-functionalised analogues shown in **Figure S4**, **Figure S5**, **Figure S19** and **Figure S23**. Arranged by modification type. Identical to **Table S6** but primarily classified by modification stage.

Sample	Source[a]	I_D/I_G	$HWHM_D$ / cm^{-1}	$I_D/I_{D'}$	A_D/A_G[b]	L_a^{TK} / nm[c]	L_a^{A} / nm[d]	L_a^{HWHM} / nm[e]	L_D / nm[f]	Ref.[g]
G	NMP	0.76	19	4.9	1.17	5.8	32	16	15	•
	C750	0.86	30	2.6	1.99	5.1	13	10	9	•,▫
	C750	1.26	35	3.2	2.77	3.5	14	9	9	◊
	C300	1.05	32	3.4	1.98	4.2	19	9	11	◊
	M25	0.39	32	2.1	1.36	11	28	9	13	◊
	CVD[h]	0.03	20	—	0.03	1.7×10^2	1.1×10^3	15	85	⌂
	CVD[h]	0.32	13[j]	—	0.21[j]	14	1.8×10^2[j]	22[j]	34[j]	⌂
	CVD[i]	0.11	17	—	0.17	41	2.2×10^2	18	38	⌂
G–SO_3^-	NMP	0.89	21	4.3	1.11	4.9	34	14	15	•
	C750	0.54	34	2.2	1.33	8.1	20	9	11	•,▫
	C750	1.19	44	4.0	3.54	3.7	11	7	8	◊
	C300	0.42	30	2.6	1.07	11	35	10	15	◊
	M25	0.18	50	5.5	1.19	25	32	6	14	◊
G–SH	NMP	0.73	21	4.1	1.26	6.0	30	14	14	•
	C750	0.69	39	2.5	1.78	6.3	15	8	10	•,▫
	CVD	0.49	16	4.0	0.70	8.9	54	19	19	⌂
G–NO_2	NMP	0.50	25	4.4	0.96	8.9	28	8	12	•
	C750	1.31	39	4.4	2.31	3.3	12	8	9	•,▫
G–NH_2	NMP	0.40	24	3.1	1.02	11	26	8	12	•
	C750	1.06	35	2.9	2.74	4.1	10	9	8	•,▫
G–Dye	CVD	0.09	28	0.5	0.11	52	3.4×10^2	11	47	⌂

[a] NMP = graphite sonochemically exfoliated in *N*-methyl-2-pyrrolidone, C750, C300, M25 = graphene/graphite powder from XG Sciences. [b] based on fitted areas (see Section S2). [c] Domain size estimate based on Eq. S1. [d] Domain size estimate based on Eq. S2. [e] Domain size estimate based on Eq. S3. [f] Estimate of average spacing between point defects based on Eq. S4. [g] Figure reference: ▫ =**Figure S4**, ⌂ = **Figure S5** , • = **Figure S19**, ◊ = **Figure S23**. [h] Raman spot focused area with no visible edges. [i] Raman spot centred on visible edge. [j] Large error associated with these value: $HWHM_D$ = (13 ± 7) cm^{-1}, L_{HWHM} = (22 ± 12) nm, A_D/A_G = 0.21 ± 0.10, L^A = (176 ± 78) nm, L_D = (33.8 ± 7.5) nm.

Table S6. Summary of Raman disorder metrics for the GNPs and their sulfur-functionalised analogues shown in **Figure S4**, **Figure S5**, **Figure S19** and **Figure S23**. Identical to **Table S5** but primarily classified by modification type.

Source[a]	Sample	I_D/I_G	$HWHM_D$ / cm^{-1}	$I_D/I_{D'}$	A_D/A_G[b]	L_a^{TK} / nm[c]	L_a^{A} / nm[d]	L_a^{HWHM} / nm[e]	L_D / nm[f]	Ref.[g]
CVD	G[h]	0.03	20	–	0.03	1.7 × 10	1.1 × 10^3	15	85	⌂
	G–Dye	0.09	28	0.5	0.11	52	3.4 × 10^2	11	47	⌂
CVD	G[h]	0.32	13[j]	—	0.21[j]	14	1.8 × 10^2[j]	22[j]	34[j]	⌂
	G[i]	0.11	17	—	0.17	41	2.2 × 10^2	18	38	⌂
	G–SH	0.49	16	4.0	0.70	8.9	54	19	19	⌂
NMP	G	0.76	19	4.9	1.17	5.8	32	16	15	•
	G–SO_3^-	0.89	21	4.3	1.11	4.9	34	14	15	•
	G–SH	0.73	21	4.1	1.26	6.0	30	14	14	•
	G–NO_2	0.50	25	4.4	0.96	8.9	28	8	12	•
	G–NH_2	0.40	24	3.1	1.02	11	26	8	12	•
C750	G	0.86	30	2.6	1.99	5.1	13	10	9	•,▫
	G	1.26	35	3.2	2.77	3.5	14	9	9	◊
	G–SO_3^-	0.54	34	2.2	1.33	8.1	20	9	11	•,▫
	G–SO_3^-	1.19	44	3.0	3.54	3.7	11	7	8	◊
	G–SH	0.69	39	2.5	1.78	6.3	15	8	10	•,▫
	G–NO_2	1.31	39	4.4	2.31	3.3	12	8	9	•,▫
	G–NH_2	1.06	35	2.9	2.74	4.1	10	9	8	•,▫
C300	G	1.05	32	3.4	1.98	4.2	19	9	11	◊
	G–SO_3^-	0.42	30	2.6	1.07	11	35	10	15	◊
M25	G	0.39	30	2.1	1.36	11	28	9	13	◊
	G–SO_3^-	0.18	50	5.5	1.19	25	32	6	14	◊

[a] NMP = graphite sonochemically exfoliated in *N*-methyl-2-pyrrolidone, C750, C300, M25 = graphene/graphite powder from XG Sciences. [b] based on fitted areas (see Section S2). [c] Domain size estimate based on Eq. S1. [d] Domain size estimate based on Eq. S2. [e] Domain size estimate based on Eq. S3. [f] Estimate of average spacing between point defects based on Eq. S4. [g] Figure reference: ▫ =**Figure S4**, ⌂ = **Figure S5** , • = **Figure S19**, ◊ = **Figure S23**. [h] Raman spot focused area with no visible edges. [i] Raman spot centred on visible edge. [j] Large error associated with these value: $HWHM_D$ = (13 ± 7) cm^{-1}, L_{HWHM} = (22 ± 12) nm, A_D/A_G = 0.21 ± 0.10, L^A = (176 ± 78) nm, L_D = (33.8 ± 7.5) nm.

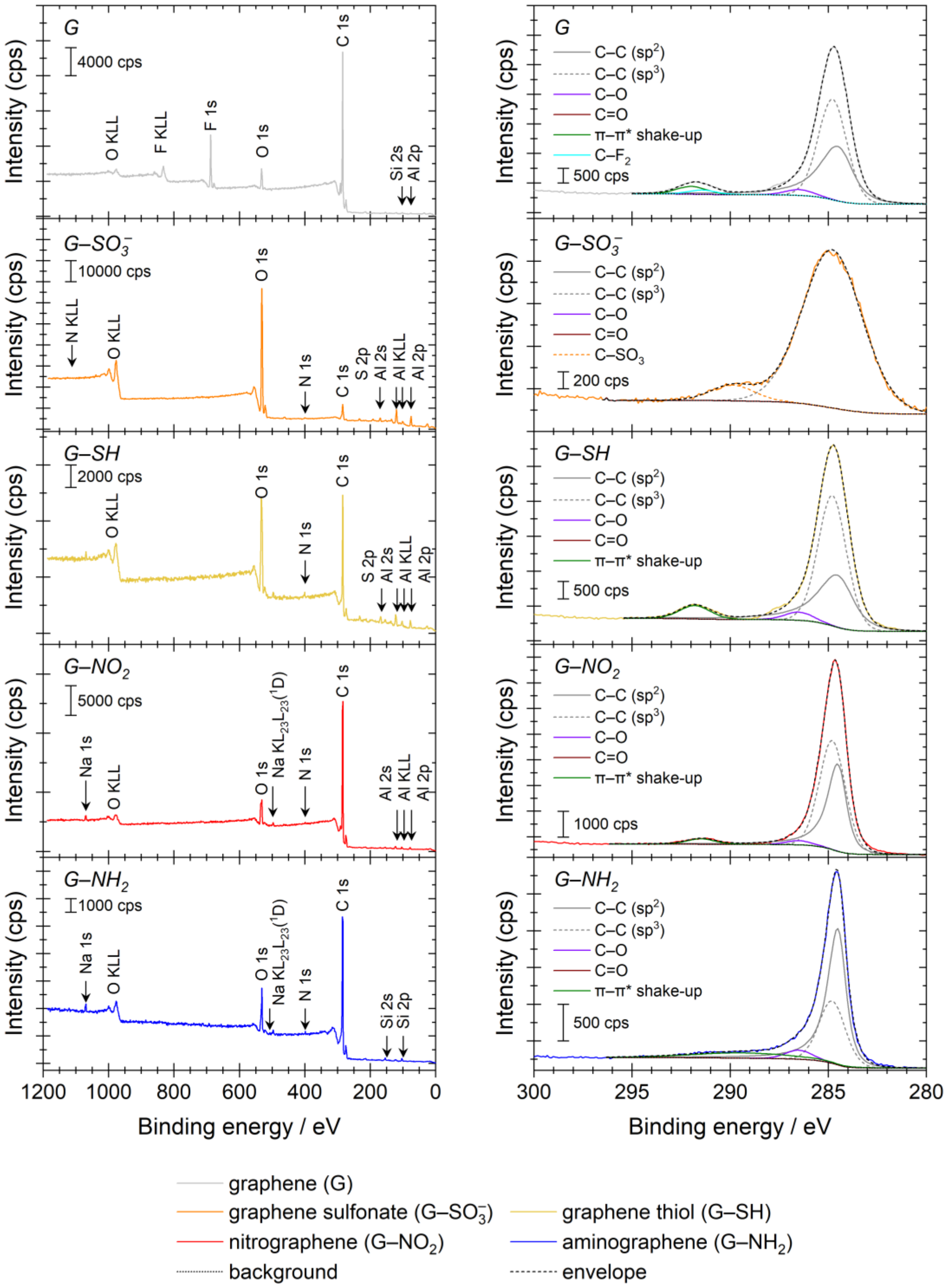

Figure S20. XPS survey scans (left column) and C 1s high-resolution scans (right column) of graphene produced by exfoliation of graphite in NMP and edge-modified analogues of them. The unmodified graphene sample has a fluorine contaminant with peaks consistent with poly(tetrafluoroethylene).

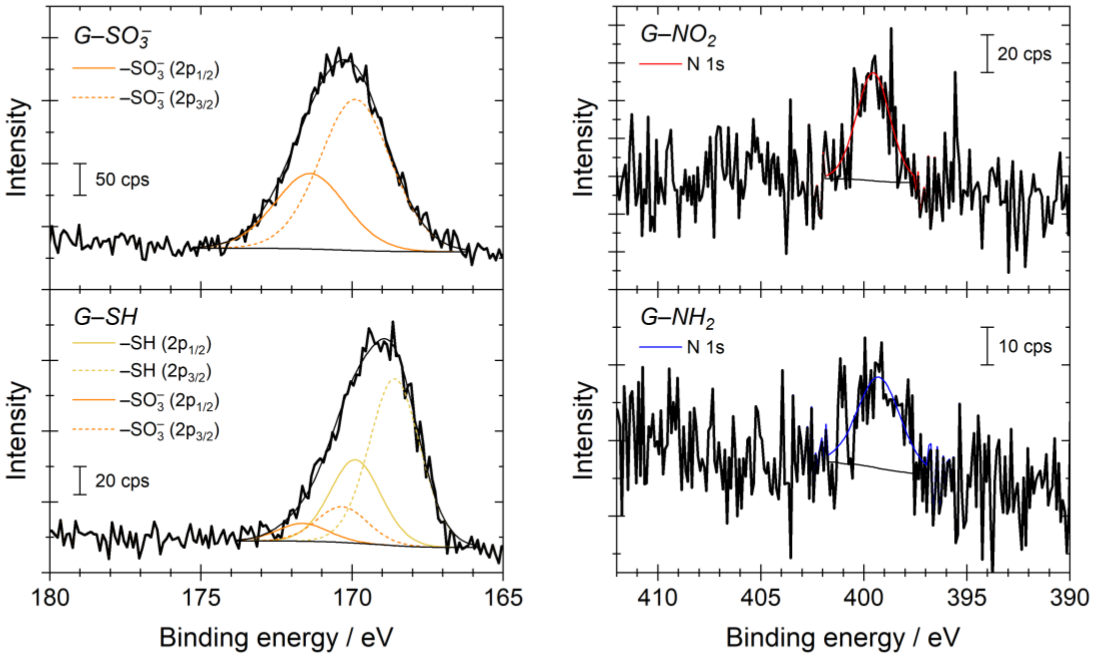

Figure S21. XPS S 2p and N 1s spectra for edge-modified graphene starting from graphite exfoliated in NMP.

S10. Effects of GNP size

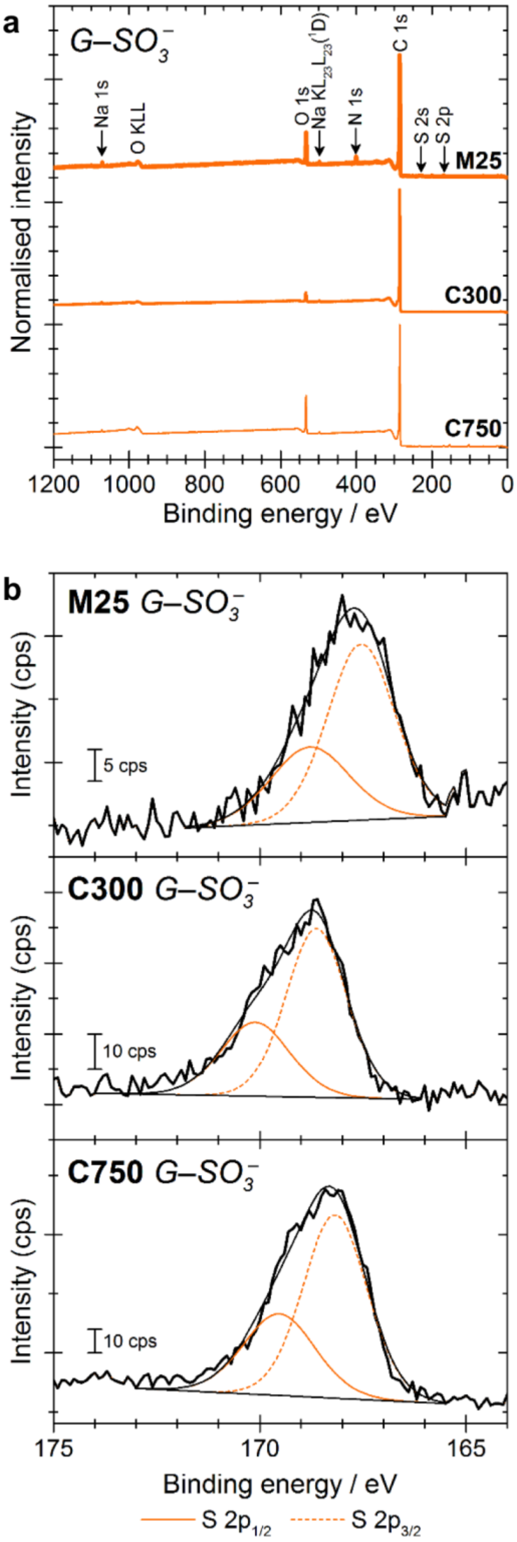

Figure S22. XPS (a) survey and (b) high-resolution S 2p spectra of three sizes of GNPs.

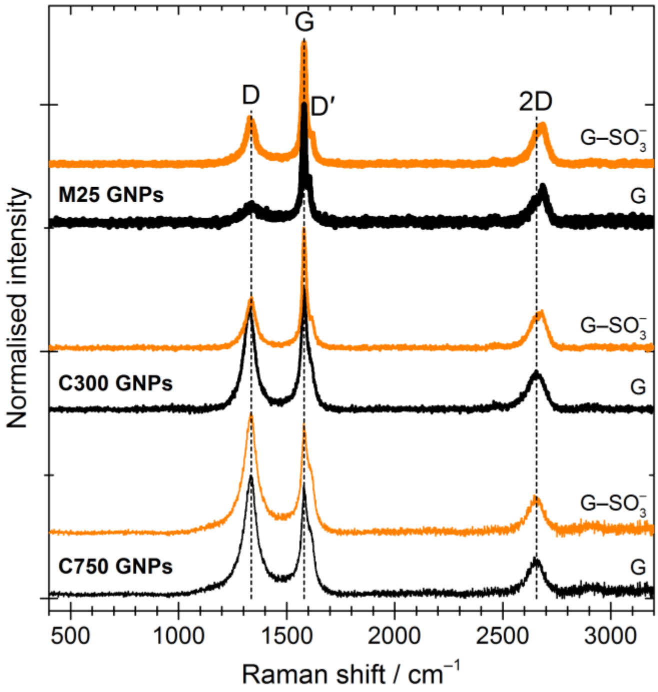

Figure S23. Comparison of the Raman spectra of three sizes of GNPs (largest on the top), and their sulfonated analogues (λ=633 nm, E_L = 1.96 eV). Three spectra were recorded for each sample and the ones with the intermediate I_D/I_G value are shown. Spectra are normalised to the most intense peak. The 2D peaks shifts to higher energy as the number of layers increases.

Table S7. Summary of the Raman peak positions for GNPs and sulfonate analogues shown as a function of GNP size.

Sample	Raman shift, D / cm^{-1}	Raman shift, max 2D / cm^{-1}
C750, G	1326	2647
C750, G–SO_3^-	1326	2647
C300, G	1325	2652
C300, G–SO_3^-	1332	2664
M25, G	1346	2677
M25, G–SO_3^-	1335	2673

S11. Zeta potential measurements

Zeta potential measurements were run on the three water-dispersible forms of edge-modified C750 GNPs: G–SO_3^-, G–NO_2 and G–NH_2. **Figure S24** shows that these samples all had negative zeta potentials, ranging between –60 mV and –50 mV. A significant negative charge was expected for G–SO_3^-, no significant charge was expected on G–NO_2 and a positive charge was expected for G–NH_2. The nitration step likely oxidised the graphene, consistent with deconvolution of the associated XPS C 1s spectra (**Table S2**), and produced charged groups similar to those found in GO. This effect was previously observed for HOPG treated in a strongly oxidising mixture of nitric acid and sulfuric acid.[9] The zeta potential for G–NH_2 will also have contributions by residual nickel oxide from the reduction of G–NO_2 with Raney nickel, consistent with measurements of the zeta potential of aqueous suspensions of nickel powders.[10]

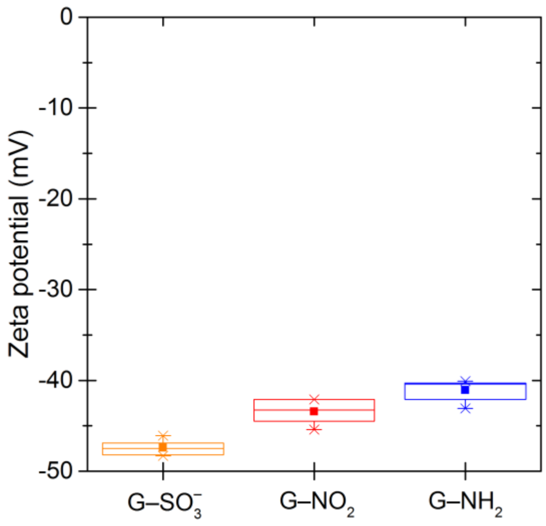

Figure S24. Box-and-whisker plots showing the zeta potential measurements for the three water-dispersible edge-modified graphene samples based on C750. (*N* = 7, box = Q2 & Q3, square = mean, cross = min/max, whisker = 1.5 × IQR)

S11. References

1. Pogorelić I, Filipan-Litvić M, Merkaš S, Ljubić G, Cepanec I, Litvić M (2007) Rapid, efficient and selective reduction of aromatic nitro compounds with sodium borohydride and Raney nickel. J Mol Catal A: Chem 274 (1):202-207. doi:https://doi.org/10.1016/j.molcata.2007.05.020
2. Puech P, Kandara M, Paredes G, Moulin L, Weiss-Hortala E, Kundu A, Ratel-Ramond N, Plewa J-M, Pellenq R, Monthioux M (2019) Analyzing the Raman Spectra of Graphenic Carbon Materials from Kerogens to Nanotubes: What Type of Information Can Be Extracted from Defect Bands? C 5 (4):art. no. 69. doi:10.3390/c5040069
3. Tuinstra F, Koenig JL (1970) Raman Spectrum of Graphite. J Chem Phys 53 (3):1126-1130. doi:10.1063/1.1674108
4. Cançado LG, Takai K, Enoki T, Endo M, Kim YA, Mizusaki H, Jorio A, Coelho LN, Magalhães-Paniago R, Pimenta MA (2006) General equation for the determination of the crystallite size La of nanographite by Raman spectroscopy. Appl Phys Lett 88 (16):163106. doi:10.1063/1.2196057
5. Puech P, Plewa J-M, Mallet-Ladeira P, Monthioux M (2016) Spatial confinement model applied to phonons in disordered graphene-based carbons. Carbon 105:275-281. doi:10.1016/j.carbon.2016.04.048
6. Lucchese MM, Stavale F, Ferreira EHM, Vilani C, Moutinho MVO, Capaz RB, Achete CA, Jorio A (2010) Quantifying ion-induced defects and Raman relaxation length in graphene. Carbon 48 (5):1592-1597. doi:10.1016/j.carbon.2009.12.057

7. Panda D, Nandi A, Datta SK, Saha H, Majumdar S (2016) Selective detection of carbon monoxide (CO) gas by reduced graphene oxide (rGO) at room temperature. RSC Adv 6 (53):47337-47348. doi:10.1039/C6RA06058G
8. Klingler RJ, Kochi JK (1981) Electron-transfer kinetics from cyclic voltammetry. Quantitative description of electrochemical reversibility. J Phys Chem 85 (12):1731-1741. doi:10.1021/j150612a028
9. Shin Y-R, Jung S-M, Jeon I-Y, Baek J-B (2013) The oxidation mechanism of highly ordered pyrolytic graphite in a nitric acid/sulfuric acid mixture. Carbon 52:493-498. doi:10.1016/j.carbon.2012.10.001
10. Hernández N, Moreno R, Sánchez-Herencia AJ, Fierro JLG (2005) Surface Behavior of Nickel Powders in Aqueous Suspensions. J Phys Chem B 109 (10):4470-4474. doi:10.1021/jp0448954
